# Supplementary material for: Leveraging protein language models and a scoring function for indel characterization and transfer learning
Source: Patterns (N Y). 2025 Nov 26;7(2):101425. doi: 10.1016/j.patter.2025.101425 (PMC12921505; doi:10.1016/j.patter.2025.101425)
Supplement: Document S2. Article plus supplemental information [file mmc2.pdf]

Patterns

Leveraging protein language models and a scoring function for indel characterization and transfer learning

Graphical abstract

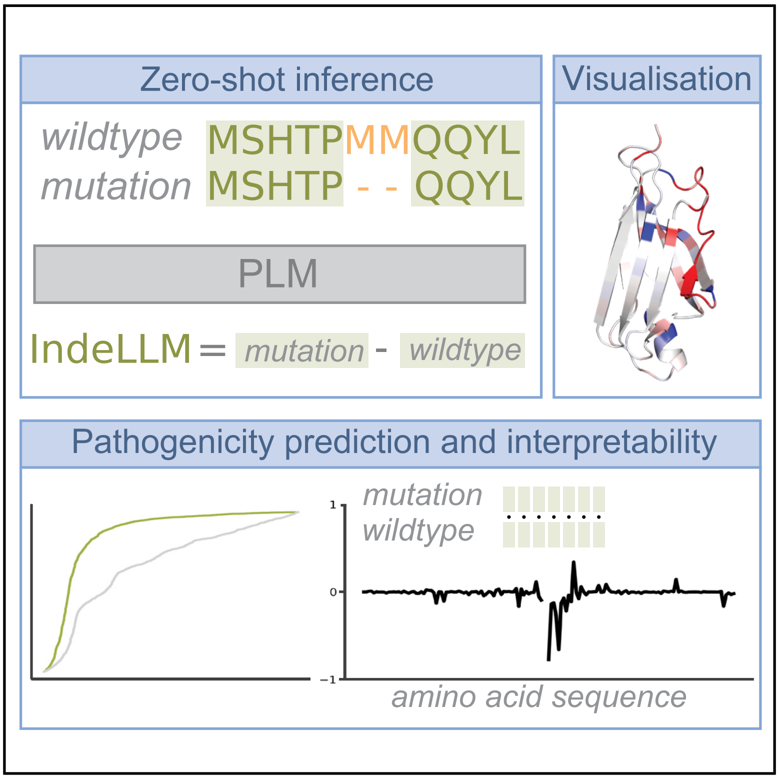

Highlights

- Improved scoring framework for indel effects using protein language models
- Increased interpretability by mapping indel impact to specific protein regions
- Improved prediction accuracy using a compact Siamese network
- Practical guidelines to enhance transfer learning for indel-focused studies

Authors

Oriol Gracia Carmona, Vilde Leipart, Gro V. Amdam, Christine Orengo, Franca Fraternali

Correspondence

o.carmona@ucl.ac.uk (O.G.C.), vilde.leipart@nmbu.no (V.L.)

In brief

Insertions and deletions (indels) are difficult to study because they change protein sequence length, limiting existing tools. Here, IndeLLM is presented: a framework that leverages protein language models to assess indel effects in a more interpretable and generalizable way. The method maps indel impact to protein regions, improves predictive accuracy with a Siamese network, and provides guidelines for transfer learning. IndeLLM offers new opportunities for indel annotation and disease-related variant analysis.

Gracia Carmona et al., 2026, Patterns 7, 101425
February 13, 2026 © 2025 The Authors. Published by Elsevier Inc.
https://doi.org/10.1016/j.patter.2025.101425

CellPress

Patterns

CellPress
OPEN ACCESS

Article

Leveraging protein language models and a scoring function for indel characterization and transfer learning

Oriol Gracia Carmona,[1,2,7,8,*] Vilde Leipart,[1,3,7,*] Gro V. Amdam,[3,4] Christine Orengo,[1] and Franca Fraternali[1,5,6]
[1]Research Department of Structural and Molecular Biology, Division of Biosciences, University College London, London WC1E 6BT, UK
[2]Randall Centre for Cell & Molecular Biophysics, King's College London, New Hunt's House, Guy's Campus, London SE1 1UL, UK
[3]Faculty of Environmental Sciences and Natural Resource Management, Norwegian University of Life Sciences, 1432 Ås, Norway
[4]School of Life Sciences, Arizona State University, Tempe, AZ 85287, USA
[5]Institute of Structural and Molecular Biology, University College London, London WC1E 6BT, UK
[6]Department of Biological Sciences, Birkbeck, University of London, London WC1E 7HX, UK
[7]These authors contributed equally
[8]Lead contact
*Correspondence: o.carmona@ucl.ac.uk (O.G.C.), vilde.leipart@nmbu.no (V.L.)
https://doi.org/10.1016/j.patter.2025.101425

THE BIGGER PICTURE Studying the effects of insertions and deletions (indels) remains a significant challenge, particularly since they alter sequence length. This issue complicates the study of indels when using high-performing protein language models (PLMs). Existing pathogenicity prediction tools are based on limited knowledge, are primarily focused on human proteins, and typically lack interpretability. We introduce an improved scoring framework that applies PLMs to studying indels. Our approach takes advantage of these models' ability to generalize across different organisms. It also enables researchers to directly map indel effects to specific protein regions, offering improved interpretability and structural insight. We also outline a set of practical guidelines for transfer learning with PLMs to enhance the efficiency and effectiveness of indel-related studies. We design a compact Siamese network, a smaller task-specific architecture combined with the framework, that outperforms current state-of-the-art indel pathogenicity predictors. This approach contributes to more robust and interpretable protein modeling, with potential implications for indel annotation, comparative genomics, and disease-related variant analysis.

SUMMARY

Protein language models (PLMs) are increasingly used to assess the impact of genetic variants, achieving high accuracy and often outperforming traditional pathogenicity predictors. They enable zero-shot inference, making predictions without task-specific fine-tuning, though studying in-frame insertions and deletions (indels) remains challenging due to altered protein lengths and limited annotated datasets. Here, we present IndeLLM, a scoring approach for indel pathogenicity that accounts for sequence length differences. Our zero-shot method relies solely on sequence information, requires minimal computing resources, and achieves performance comparable to existing predictors. Building on this, we developed a Siamese network via transfer learning that outperformed all tested indel predictors (Matthews correlation coefficient = 0.77). To enhance accessibility, we provide a plug-and-play Google Colab notebook for using IndeLLM and visualizing the impact of indels on protein sequence and structure. The tool is freely available on GitHub and Google Colab.

INTRODUCTION

Insertion and deletion variants (indels) are among the most prevalent forms of genetic variation, accounting for about 18% of all variation in humans, with individuals sometimes carrying hundreds of these mutations across their exome.[1,2] These variations are commonly categorized into in-frame indels, which add or remove entire codons without disturbing the reading frame, and frameshift indels, which shift the reading frame by adding or removing nucleotides in non-triplet amounts, producing entirely

This is an open access article under the CC BY license (http://creativecommons.org/licenses/by/4.0/).

new downstream protein sequences. The effects of indels can vary significantly, with some leading to substantial changes in protein structure and function, while others remain largely benign.[3,4]

Frameshift indels, which disrupt the reading frame and typically result in loss of protein function, are generally considered pathogenic.[5] Here, we are considering in-frame indels, which, in contrast, preserve the reading frame while altering the amino acid sequence. These indels can potentially impact protein stability and function. Their effects are less predictable than frameshift indels and are often comparable to those of missense variants.[6]

Although in-frame indels are known to cause several well-documented monogenic diseases, such as cystic fibrosis, eye disorders such as childhood cataracts and retinal dystrophies, and several types of human cancers,[5–7] their pathogenicity has historically been less studied than that of single-nucleotide variants (SNVs).[1,8]

As a result, the mechanism behind pathogenicity for in-frame indels is far from understood. Despite this, some specialized computational approaches are available to predict in-frame indel pathogenicity. The tools employ diverse strategies, often relying on the use of several manually encoded predictive features, such as sequence conservation, indel size, and protein structure and function, that have been seen to correlate with pathogenicity.[8–12] However, selecting such features is based on an incomplete knowledge of the underlying mechanisms. It may not accurately capture the full complexity of indel pathogenicity or may potentially introduce biases that emphasize certain aspects while overlooking others. In addition, most available tools are often tailored to the human genome, as creating more broadly applicable tools is challenging due to limited available pathogenicity annotations for indels from other species.

Protein language models (PLMs) have the ability to meet these challenges. These models have emerged as a transformative approach in artificial intelligence, particularly for specialized fields like protein sequence analysis. PLMs are able to capture intricate relationships in proteins only by using protein sequences as input through training on massive unlabeled protein sequence datasets. The obtained embeddings contain rich biological information, which can be used as input for transfer learning approaches. PLMs offer a complementary technique to those based on genomic features and can be used to infer the potential effects of variants at the protein level.

These PLMs can capture complex information about protein secondary and tertiary structures across a wide range of organisms, from bacteria to humans, using the amino acid sequence as its only input feature, potentially providing insights beyond those available from traditional methods at a faster speed. Advances in computational power and the growth of available protein sequences drive the field of PLMs to create models with advanced transformer architectures containing billions of parameters. Today, a diverse range of PLMs, varying in size and architecture, is available, from general-purpose to specialized models.[13–18]

Transformer-based architectures and PLMs have shown great success and revolutionized the SNV prediction field,[13,19,20] both when used as zero-shot inference methods (using the predicted logits with no additional training[21]) and when fine-tuned or used as features for other models. Zero-shot inference approaches are appealing since they do not risk overfitting to the data. However, when dealing with indels, using these techniques is not straightforward because of the difference in length between the sequences, which adds extra challenges.[19,22] Recent efforts have focused on developing methods leveraging PLMs, either as zero-shot inference tools using a generalized scoring function described by Brandes et al.[19] or as features to train other downstream models.[9,23] However, these approaches still have limits in their accuracy or possible use cases. For example, SHINE, an elastic net model trained on a principal-component decomposition of PLM embeddings, is limited to small in-frame indels of one or two amino acids and requires two independent models, one for deletions and one for insertions.[23]

Despite these efforts, it remains unclear which PLMs provide the most accurate latent representations of the protein effects caused by indels, how best to transfer those representations, which scoring functions perform best for zero-shot indel prediction, and what the limitations of these methods are. Furthermore, the interpretability of predictions from PLMs can be challenging, as the patterns they capture are often complex and not immediately intuitive.[24,25]

In this work, we present an in-depth analysis of the inner workings of general scoring functions for zero-shot pathogenicity classification for in-frame indels. We suggest an improved scoring regime that achieved an improvement of 0.07 points in the Matthews correlation coefficient (MCC) over a general scoring approach,[19] reaching an MCC comparable to the best-performing methods for indel prediction without any further training. From there, we test different transfer learning approaches on a simple one-hidden-layer Siamese network and manage to obtain unprecedented performance through a biology-guided slicing of the PLM embeddings. Finally, we present an analysis approach that aids in understanding the model predictions by looking at the local effects of the indel on the rest of the protein amino acids.

RESULTS

Efficient scoring approach for zero-shot inference using protein language models

Our dataset has 7,500 indels, of which 2,409 are insertions and 5,091 are deletions. Of these, 2,878 are classified as likely pathogenic or pathogenic, while 4,622 are classified as likely benign or benign. We used the dataset to assess different scoring methods for the zero-shot inference using PLMs. The PLM of choice to test the scoring methods was ESM2 (650M parameters),[15] which has shown the best performance compared with the other tested PLMs (Figure S3). Using PLMs as zero-shot predictors is not straightforward due to the varying lengths of both the sequences and the indel sizes.[22] Brandes et al.[19] described a generalizable approach to score sequences using the pseudo-log likelihood (PLL) of each sequence calculated as the sum of the log probabilities of each observed amino acid (see methods for more details). The described general approach, referred to as Brandes scoring, achieved a reasonable performance of MCC 0.58 (Figures 1A and 1B).

One concern of such a scoring method is that the PLLs being compared are not comparable due to the difference in length in the wild-type and mutated sequences. To mitigate this, we

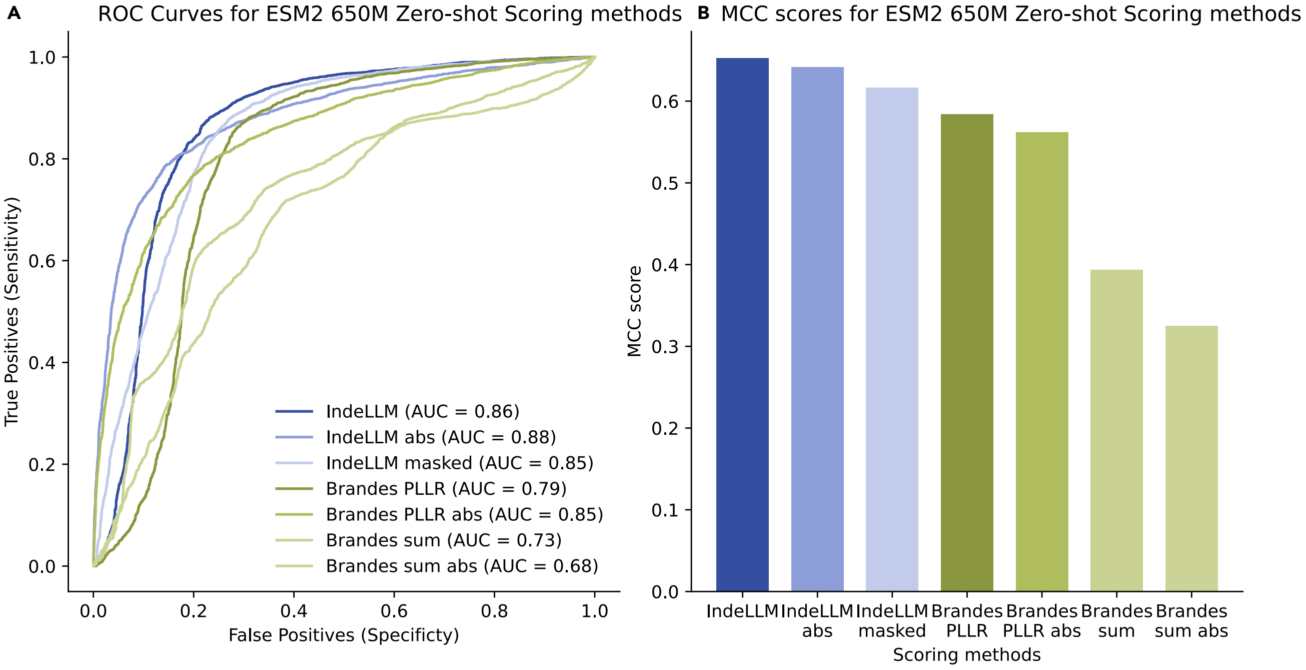

Figure 1. Comparison of zero-shot scoring methods for ESM2

Evaluation of different zero-shot scoring methods using the probabilities of ESM2 (650M parameters). The different scoring methods were IndeLLM (probabilities of overlapping regions), IndeLLM abs (using the absolute probability of overlapping regions), IndeLLM masked (logits from masked positions), Brandes (sum of all probabilities), and Brandes abs (using the absolute sum of probabilities). We compared the scoring methods by (A) plotting the ROC curve and calculating the AUC and (B) calculating the MCC scores. See Table S1 for all scores.

removed the probabilities of the deleted or inserted amino acids, calculating a PLL for the overlapping regions. Doing this eliminates the issue of incomparable likelihoods by indirectly inferring the indel's effect. Another concern is that of fluctuations in probabilities within less-conserved regions. Here, the PLM assigns low probabilities to both the wild-type and the mutant sequences, which can disproportionately influence the final score when using methods such as log-probability summation or direct probability multiplication (Figure S2). To mitigate this effect, we propose using the sum of probabilities instead. This simplified scoring approach, which we denote as IndeLLM, produced an improvement in the predictions, ranking first among the tested scoring methods (Figures 1A and 1B; Table S1).

Another factor to consider is the impact of the indels, which significantly increase the likelihood of a protein being potentially pathogenic. To account for that, the absolute value of the difference between PLLs or scores is used instead. However, this scoring method, denoted as IndeLLM abs or Brandes abs, showed no improvement compared with using just the plain subtraction of PLLs or scores (Figures 1A and 1B).

Finally, we also compared two different ways to calculate the marginal probabilities, one using a single encoding step and then using that to compute the logits at each position (IndeLLM). The other uses a masking objective by masking each position individually and then using the logits of the masked position only (IndeLLM masked). Both approaches showed marginal differences in performance (Figures 1A and 1B; MCC IndeLLM 0.65 vs. IndeLLM masked 0.62), with the single encoding approach requiring significantly less computation time.

Leveraging transfer learning and Siamese networks for indel prediction

We leveraged PLMs in combination with a Siamese network to predict the pathogenicity of indels. Since PLMs are pre-trained models, we can extract high-quality embeddings that encode the structural and functional properties of sequences without requiring extensive task-specific training. These embeddings were fed into a smaller, task-specific architecture, a Siamese network, to efficiently compare and analyze sequence pairs.

Due to the small size of the labeled indel dataset (n = 7,500), we used a small multilayer perceptron (MLP) model with only one hidden layer. This mitigated overfitting by limiting the model's complexity and, thus, its capacity to memorize the training data by picking up patterns in the noise that are not generalizable to the populations. In particular, using small Siamese networks for pathogenicity prediction has shown high performance in single-point mutation pathogenicity prediction.[20]

We used 80% (n = 5,960) of the labeled indels for the training dataset, while the remaining indels were split into a validation and a test dataset (n = 819 and n = 721, respectively). A robust model should be able to predict the pathogenicity of unseen protein sequences and indels. Therefore, it is essential to avoid redundancy between training, test, and validation datasets (data leakage). We clustered all wild-type protein sequences by sequence identity above 50% and randomly divided the clusters into the three datasets, achieving a maximum of 50% identity between all three datasets. We also ensured that the distributions of indel type (insertions or deletions), indel size (short [1 or 2 amino acids] or long [$\geq$3 amino acids]), and indel classification

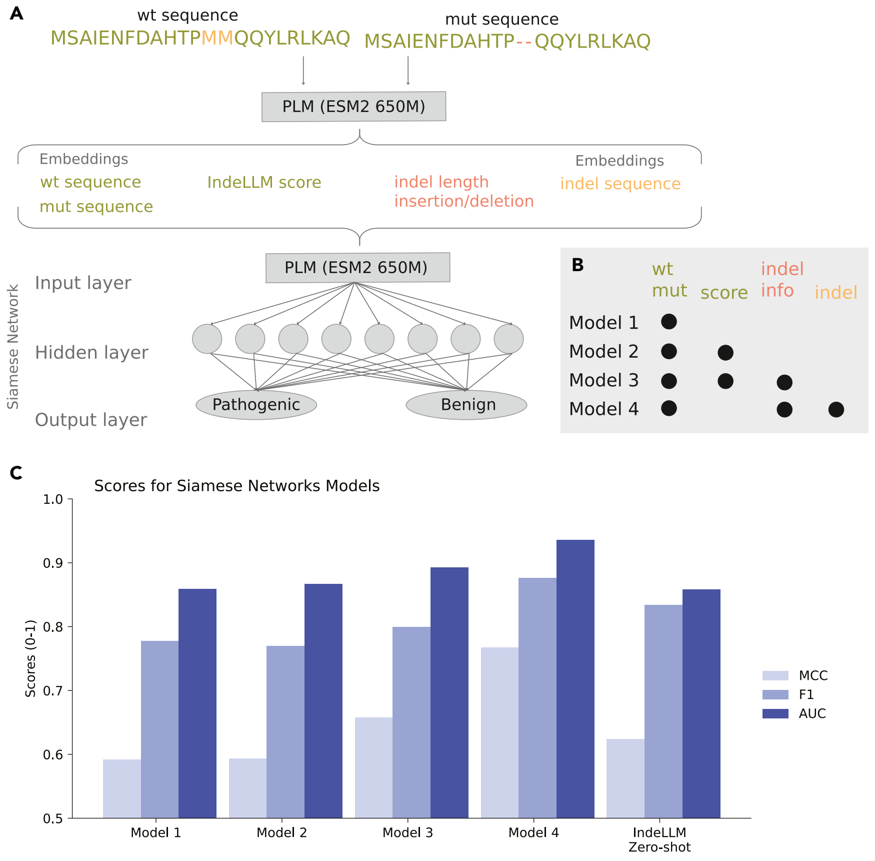

Figure 2. Siamese network architecture and performance

(A) Siamese network setup. From the wild-type and mutated sequences, we generated four types of weights: the mean embeddings for the wild-type and mutated sequences (the full sequences), the IndeLLM score (using only overlapping regions, colored green), the indel type and length (based on the gap position and size, colored red), and the mean embeddings of the inserted or deleted amino acids (indel sequence, colored orange). The setup includes only one hidden layer of eight nodes.

(B) Table of the weight used in models 1–4 marked with black dots and the same coloring as in (A).

(C) The MCC (light blue), F1 (blue), and AUC (dark blue) scores for the mean of models 1–4 and IndeLLM Zero-shot using the test dataset.

(pathogenic and benign) were equal to the distributions in the complete dataset (see methods for details).

We used a setup similar to the one suggested by VariPred,[20] namely, a Siamese network with one hidden layer, using a LeakyReLu as an activation function and a dropout rate of 0.5. Other hyperparameters, such as the number of neurons in the hidden layer, batch size, learning rate, and negative slope parameter for the LeakyReLu, were optimized through a simple grid search (Figure 2A).

The difference in length between the wild-type and the mutated sequences adds an extra layer of complexity when deciding which part of the embedding to use as input. Other approaches using PLM embeddings as input features have solved this by separating insertions from deletions.[23] Here, we tested four different Siamese architectures to find which approaches provide the best transfer of the relevant latent space (Figures 2A and 2B).

The four models are variations of a Siamese network designed for sequence analysis. Model 1 utilizes mean embeddings from the last hidden layer for both wild-type and mutant sequences. Model 2 builds on this by incorporating the IndeLLM score as an additional input. Model 3 further extends this by including the indel type (insertion or deletion) and length as extra inputs. Model 4 splits the embeddings into overlapping and non-overlapping (indel) regions while also integrating indel type and length as inputs.

All replicates for model 1 using a more classical representation of the wild-type and mutant sequences by extracting the mean embeddings performed equal to the IndeLLM score in terms of area under the curve (AUC) of the receiver operating characteristic (ROC) curve but generally underperformed in terms of MCC and F1 score (performance on the test dataset was AUC, 0.86 vs. 0.86; MCC, 0.59 vs. 0.62; and F1, 0.78 vs. 0.83, respectively), indicating that the mean embeddings did not contain unique information when compared with the proposed IndeLLM score. Adding the IndeLLM score, model 2, did not improve the performance (MCC, 0.59). Model 3 was the only model from the group with an MCC comparable to the IndeLLM score (0.66 vs. 0.62, respectively), suggesting that providing the indel length and type contributed information on top of the plain mean embeddings. Model 4 performed significantly better than the IndeLLM score and any of the other three models, achieving MCC performance on the test set of 0.77, a 24% improvement from the IndeLLM score. This suggests that the information captured by the embeddings of the inserted or deleted amino acids adds additional information to the sequence embeddings. A more detailed breakdown of the performances of each model on each of the available datasets can be found in Table S2.

Comparing indel predictor performance with the IndeLLM scoring approach and Siamese model

We used the scores of nine different tools published by Cannon et al.[9] and evaluated their performance and indications of overfitting (Figures S4 and S5). We concluded that the best-performing indel pathogenicity prediction tools were Provean and MutPred-indel, which were used to obtain pathogenicity prediction for all 7,500 indels. We compared performance using the IndeLLM Zero-shot score to the Provean and MutPred-indel on all indels (n = 7,500), the training (n = 5,960), validation (n = 819), and test (n = 721) datasets. We observed consistent pathogenicity prediction performance for all methods (Figure 3). MutPred-indel and IndeLLM have comparable performance (MCC MutPred-indel 0.57 vs. MCC IndeLLM 0.62).

CellPress
OPEN ACCESS

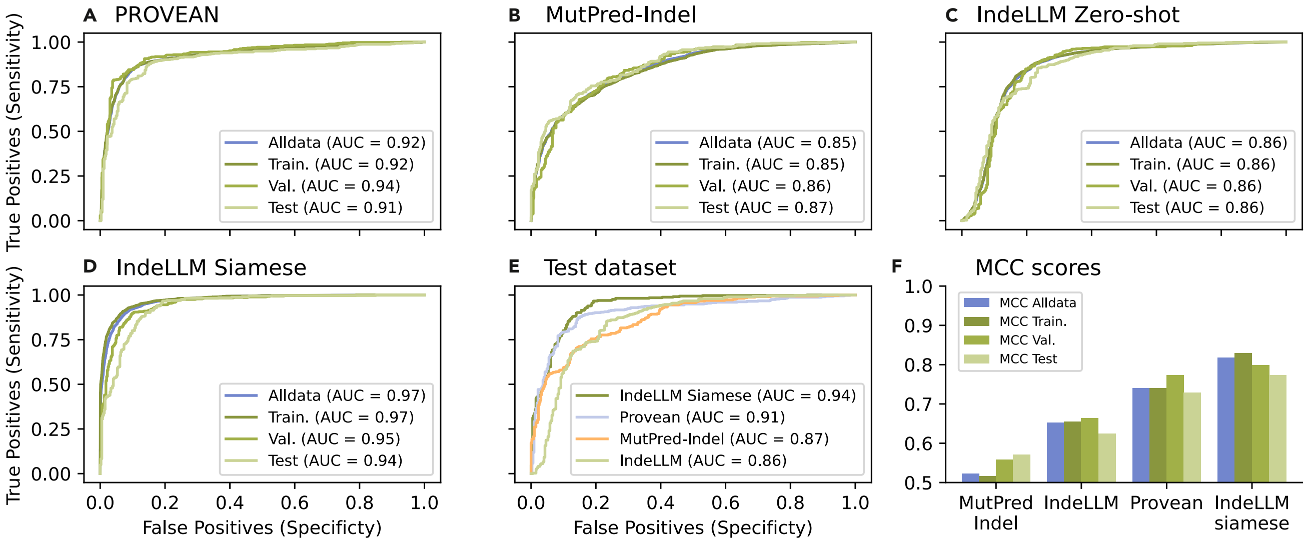

Figure 3. Performance comparison of IndeLLM and pathogenicity prediction tools across datasets
Evaluation of the performance of the best-performing pathogenicity prediction tools, (A) Provean and (B) MutPred-indel, compared with (C) IndeLLM Zero-shot and (D) IndeLLM Siamese on all collected indels (blue) and the different dataset splits (green) used for training (train.), validation (val.), and testing (test). In (E), we compare their performance on the test dataset. In (A)–(E), we plotted the ROC curve and calculated the AUC, while in (F), we show the MCC scores for each method per dataset grouping and sorted by the MCC on the test data split.

IndeLLM Siamese outperformed all methods and has 5.5% better prediction accuracy than the best-performing prediction tool, Provean (MCC IndeLLM Siamese 0.77 vs. MCC Provean 0.73) (Figure 3F). See Table S3 for all scores.

The Siamese network outperformed the zero-shot inference approach by 24%. To assess the potential for overfitting, we evaluated the model on a holdout dataset derived from a recent iteration of ProteinGym, excluding any variants previously included in the curated training set. IndeLLM Siamese maintained comparable performance on this independent set, achieving an AUC of 0.90 and an MCC of 0.79 (Figures S5 and S6; Tables S4 and S5).

Examining the confusion matrix (Figure 4) reveals a high reduction in false negatives for insertions (19.51% for IndeLLM Zero-shot and 6.10% for IndeLLM Siamese) (Figures 4B and 4D). For the IndeLLM score predictions, we observe that the false-negative predictions for insertions have a high mean probability for the inserted amino acid, similar to the real benign insertions (Figure S7). Additionally, these false-negative insertions tend to be longer than benign insertions. However, this effect cannot be observed using the IndeLLM Siamese model. This suggests that some insertions may not introduce damaging amino acids but may instead cause pathogenicity through increased stability or altered fit, which the IndeLLM Zero-shot scoring approach fails to detect. By incorporating sequence embeddings for the inserted or deleted amino acids in Siamese model 4, we were able to account for these instances and accurately predict pathogenicity.

Toward interpretable indel predictions

Interpretability is essential for the usability of machine models. Understanding how these models predict changes in protein fitness allows users to validate results, ensuring that the predictions align with biological principles and are not artifacts or model biases.

The zero-shot scoring method by PLMs per amino acid allows us to obtain information on the consequences of the indels for the protein environment. The model assigns a value to each amino acid in the sequence based on its predicted likelihood given the rest of the sequence, which indicates the evolutionary patterns. By subtracting the per-amino-acid wild-type probabilities from the per-amino-acid mutated probabilities (except the deleted or inserted amino acids) for the entire protein sequence, we obtain a difference in value per amino acid. The differences range from 1 to -1, where 1 can be interpreted as the indel introducing beneficial consequences for the amino acids while -1 is detrimental. By plotting the differences in amino acid values, we can visualize the impact of the indel on the protein environment and, therefore, gain a better insight into why the indel is predicted as pathogenic or benign.

Our dataset reports four indels for a single protein, fibroblast growth factor receptor 1 (FGFR1). FGFR1 is a cell-surface membrane receptor built of three extracellular immunoglobulin (Ig)-like domains (D1–D3), a short hydrophobic transmembrane region, and a cytoplasmic tyrosine kinase domain.[26] Of the four indels, one is in the tyrosine kinase domain, and three are in the extracellular Ig-like domains (Table 1). The differences in amino acid probabilities illustrate which regions the indels impact (Figures 5A–5D). The two pathogenic deletions have a negative effect on the surrounding regions, while the two benign indels do not affect the protein. By coloring the 3D model by the difference, we provide an explanation for the prediction. Here, we show that deleting a methionine in position 535 is predicted to destabilize the αC helix (Figure 5E), a structural element essential for the activation of the enzyme.[26] We also found a five amino acid deletion in the first extracellular Ig-like domain (D1) in FGFR1. The long deletion occurs in a loop region, and IndeLLM predicts a destabilization and rearrangement of the domain, since we observe several interactions predicted to be

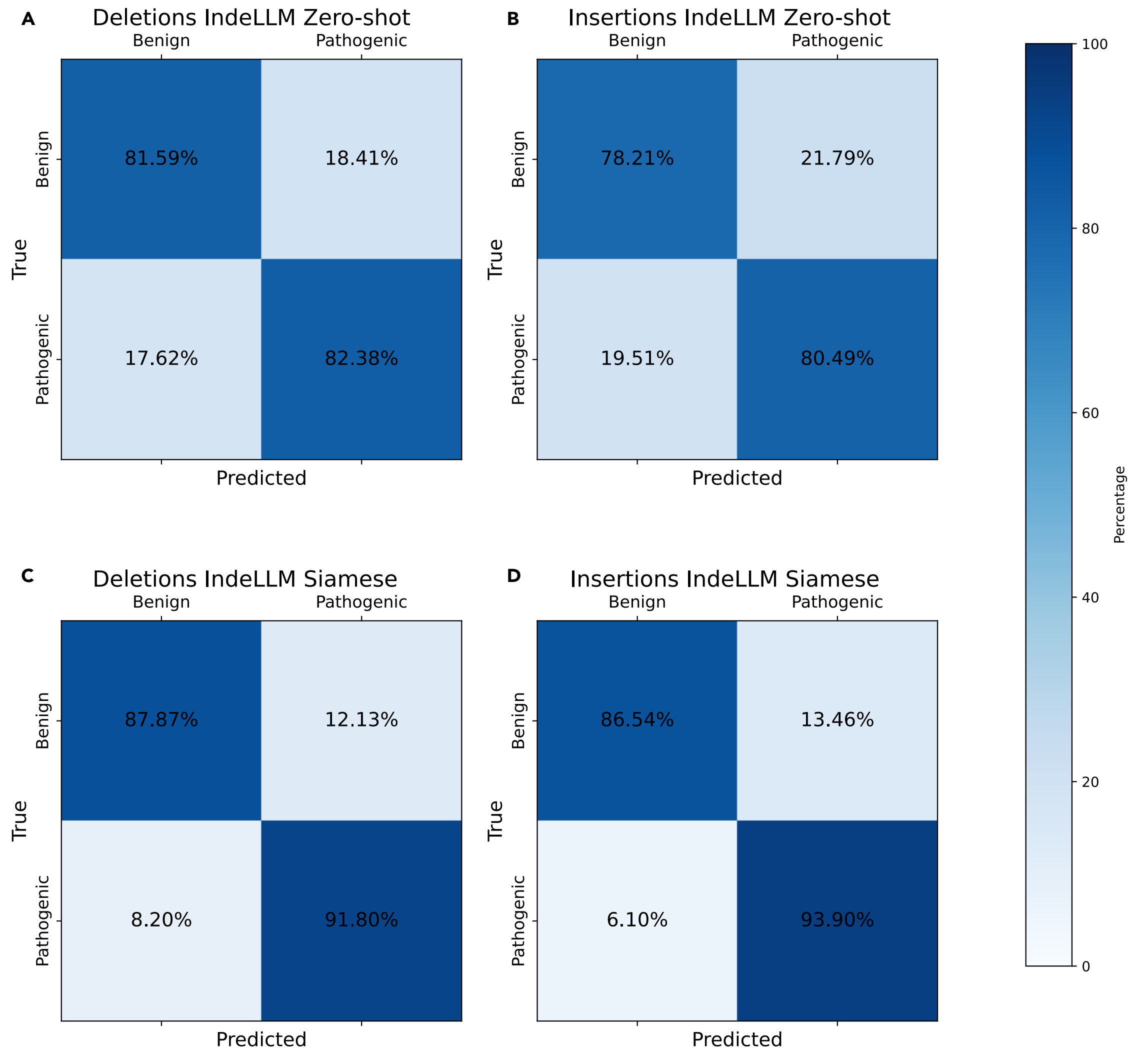

Figure 4. Confusion matrices for IndeLLM Zero-shot and Siamese models

(A) Confusion matrix for all deletions using IndeLLM Zero-shot scoring.
(B) Confusion matrix for all insertions using IndeLLM Zero-shot scoring.
(C) Confusion matrix for all deletions using IndeLLM Siamese network.
(D) Confusion matrix for all insertions using IndeLLM Siamese network.
All confusion matrices are colored by the percentage of true positives for benign (left) and pathogenic (right) insertions or deletions.

altered (lost interactions in red and new interactions in blue, Figure 5F). We tested our hypothesis by predicting the structure of the mutated domain using AlphaFold,[27] which also predicts an unfolding of the domain (Figure S8).

Next we considered the effects of indels in glomulin (GLMN), which is essential for normal vasculature development and is part of a large protein complex (GLMN-RBX1-CUL1 complex).[28] GLMN (HEAT-like) adopts a repeated fold of α helices organized in the N- and C-terminal domains. Our dataset reports two indels in the GLMN protein (Table 2). The pathogenic deletion is in the C-terminal domain, a domain that is essential for interactions with subunits in the complex.[28] The difference in probabilities

Table 1. Example of indels reported for the FGFR1 protein (UniProt: P11362)

Protein	Indel type	aa pos	FGFR1 domain	WT sequence	Mut sequence	Indel size	ClinVar	IndeLLM
FGFR1	deletion	535	tyrosine kinase domain	MK	K	1	pathogenic/likely pathogenic	pathogenic
FGFR1	insertion	133	disordered	D	DD	1	likely benign	benign
FGFR1	deletion	132	disordered	DD	D	1	likely benign	benign
FGFR1	deletion	92	Ig-like D1	VPADSG	G	5	likely pathogenic	pathogenic

CellPress
OPEN ACCESS

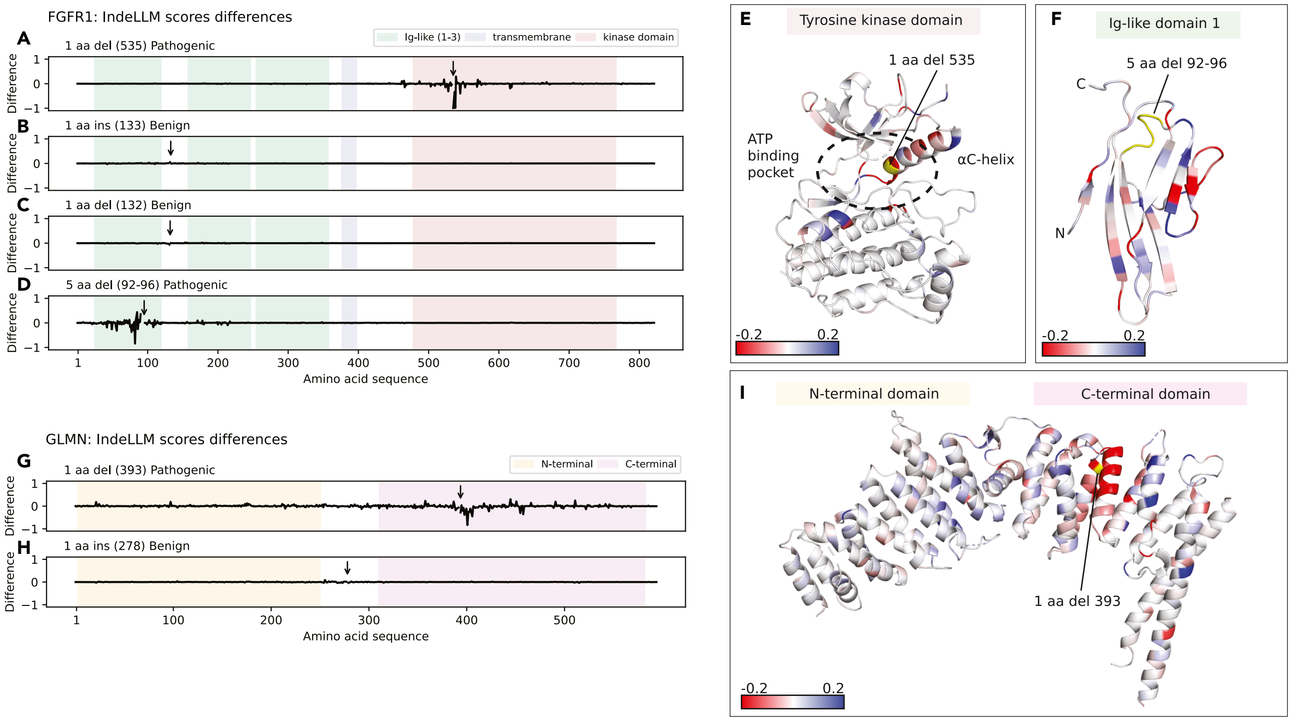

Figure 5. Mapping sequence probability differences onto protein domains and structures

(A–D) The differences between the wild-type and the mutated sequence probabilities (*y* axis) are plotted per amino acid in FGFR1 (*x* axis). The colored boxes on the plots represented the domains in FGFR1 (D1–D3 in green, transmembrane region in blue, and the tyrosine kinase domain in red). In each plot, the indel position is labeled with an arrow and the indel type is noted in the title.

(E) PDB structure of the tyrosine kinase domain (PDB: 4UWY) colored by the difference score (from red [≤ -0.2] to blue [≥ 0.2]). The indels are labeled and colored yellow. The impacted structural element (αC helix) is labeled, as well as the ATP binding pocket (dotted circle).

(F) PDB structure of the Ig-like domain 1 (PDB: 2CR3) using the same coloring scheme as in (E). The N and C termini are labeled.

(G and H) The plots are the same as in (A)–(D) but for the GLMN protein sequence. The colored boxes represented the N-terminal (yellow) and C-terminal (pink) domains.

(I) PDB structure of the GLMN protein (PDB: 4F52) is colored as in (E). The N- and C-terminal domains are labeled in colored boxes above the structure.

between the wild-type and the mutated sequences demonstrates that the deletion of asparagine in position 393 destabilizes large regions of the protein, particularly in the C-terminal domain (Figures 5G and 5I). In contrast, the benign insertion downstream of the N-terminal domain has minimal implications for the protein (Figure 5H).

DISCUSSION

We have presented here a framework for the prediction of indel scores in the context of zero-shot inference. By scoring only amino acids that are identical in the wild-type and mutant sequences, previous limitations caused by indel-induced length differences were overcome by our method. Specifically, without length correction, pathogenicity scores were heightened for deletions and decreased for insertions. This difference originates from the extra amino acids in either the mutant or the wild-type sequence, making scores not directly comparable.

To address these issues, a zero-shot scoring approach that resolves the bias introduced by non-comparable PLLs has been introduced here. Our method utilizes the context awareness of PLMs, which capture sequence dependencies and evolutionary relationships. Rather than including the inserted or deleted amino acids in the PLL calculation, we compute the scores using only the amino acids present in both wild-type and mutant sequences. By using only overlapping regions, one removes length biases, decreases noise, and ensures a balanced indel pathogenicity scoring. Given the limited availability of labeled data for indels, this represents a promising approach, as it allows for accurate pathogenicity assessment without overfitting to known cases.

Moreover, we also propose a simplified zero-shot scoring approach utilizing the sum of probabilities instead of the sum

Table 2. Example of indels reported for the GLMN protein (UniProt: Q92990)

Protein	Indel type	aa pos	GLMN domain	WT sequence	Mut sequence	Indel size	DDD	IndeLLM
GLMN	deletion	393	C-terminal domain	NK	K	1	pathogenic	pathogenic
GLMN	insertion	278	N-terminal domain	–	E	1	likely benign	benign

of log probabilities or multiplication of individual probabilities. This modification addresses a key issue: when relying on log sums or probability products, small changes in positions with already low likelihoods, typically found in poorly conserved regions,[15] can disproportionately influence the overall score. This happens because a small absolute change in a low probability corresponds to a large fold change, resulting in an exaggerated impact on the final score. In contrast, larger absolute changes at well-conserved positions, which have higher baseline probabilities, may result in smaller relative fold changes and thus contribute less to the score under those methods, despite being potentially more biologically meaningful. By summing raw probabilities, our method reduces this imbalance, ensuring that variations in high-confidence regions are not overshadowed by noise in low-confidence ones. This scoring scheme, which we term IndeLLM Zero-shot, offers a more balanced evaluation of mutation effects. IndeLLM Zero-shot scoring substantially improved the predictions, boosting PLMs from 0.58 to 0.65 (MCC score), levels comparable to those of methods trained with labeled data and manually curated features. Furthermore, we anticipate that as PLMs continue to evolve, the performance gap between zero-shot and supervised approaches will continue to narrow. In addition, our scoring strategy offers a context-aware method for evaluating indels with potential applications that extends beyond variant effect prediction in areas such as multiple sequence alignment.

One appealing use of PLMs is their ability to generate latent representations as input features for downstream models. Various strategies have been proposed to integrate PLM embeddings across applications, including the prediction of indels.[19,23] Ideally, such models should maintain simplicity to minimize the risk of overfitting when working with limited datasets. In this study, we combined the approach described in IndeLLM Zero-shot with a lightweight Siamese network consisting of a single fully connected hidden layer with eight nodes. Despite its simplicity, this approach achieved state-of-the-art performance with no evidence of overfitting (IndeLLM Siamese vs. Provean AUC: 0.94 vs. 0.91). To better understand the contributions of specific features, we conducted an ablation study, revealing that splitting the embedding of indel amino acids from the rest of the sequence embedding significantly improved performance. This highlights the importance of integrating our biological knowledge when designing the model and during feature engineering. By leveraging this understanding, we can isolate the most informative parts of the embeddings, preventing signal dilution and reducing noise while preserving rich sequence representations. This key modification enhanced model performance without adding complexity. The proposed framework, termed IndeLLM Siamese, outperformed previous approaches in classification accuracy (MCC 0.77) and demonstrated greater computational efficiency compared with previously suggested methods (Note S8). These findings indicate that the IndeLLM Siamese model can serve as a valuable tool for the community, both as a high-performing predictor and as an example of effective feature selection in transfer learning. We suggest that incorporating more biological knowledge during feature selection could further enhance variant effect prediction in a more controlled and precise manner.

A closer examination revealed that the improvements in the IndeLLM Siamese, compared with the IndeLLM Zero-shot scoring, come from the reduction in the insertions that were wrongly predicted as benign (false negatives). The confusion matrix for IndeLLM reveals a high false-negative rate for insertions (19.51%), suggesting a systematic error in the predictions for insertions. The majority of the false-negative predictions for insertions involved amino acids with higher estimated probabilities than the average observed for pathogenic insertions (Figure S7). These findings suggest that such insertions could be causing a pathogenicity effect due to increased stability or altered fit or, perhaps, gain of function.[19] IndeLLM Zero-shot scores assess the potential damage caused by the indel on the protein sequence and use that as a proxy for pathogenicity. This can explain why gain-of-function cases are not captured properly. We mitigated this by adding information about the inserted or deleted amino acids in IndeLLM Siamese model 4 (Figure 2). The negative rate for insertions dropped drastically to 6.10%. By comparing the mean inserted probabilities of the false-negative predicted insertions from IndeLLM Zero-shot and IndeLLM Siamese model 4, we observed a drop from 110 cases to 3 cases (Figure S7). This suggests that the IndeLLM Siamese model can better capture gain-of-function cases by leveraging embeddings of inserted amino acids. However, further improvements are needed to refine this capability. Mapping embeddings to identify key regions of interest presents a promising direction for future advancements.

Finally, we present an approach to interpreting the predictions by examining individual amino acid probability changes. The obtained differences can then be mapped back to the structure to spot regions predicted to be affected by the indels. This allows experts to use this information in terms of structure and function.

The Siamese network and zero-shot scoring approaches introduced in IndeLLM provide a low-complexity, biologically informed framework that can be seamlessly integrated into standard genomic analysis pipelines. These methods are particularly useful for evaluating pre-selected variants of uncertain significance (VUSs), offering insights into their potential functional impact at the protein level. Because both approaches rely solely on protein sequence information, they serve as a valuable complementing tool to existing techniques that leverage genomic or population-level data. Depending on the analysis stage and the specific type of variants under investigation, threshold values can be adjusted to better balance false-positive and false-negative rates, allowing the approach to be tailored to different research or clinical priorities.

While the developed IndeLLM Siamese network achieved the highest performance, it requires labeled training data, limiting its applicability primarily to human sequences. However, the underlying design principles can be extended to future models. In contrast, the IndeLLM Zero-shot approach offers broader applicability and ease of use, albeit with a trade-off in predictive accuracy.

In particular, the IndeLLM Zero-shot method operates at the per-position level, enabling preliminary identification of potential mechanisms of disruption, such as altered motifs or conserved regions, without requiring labeled training data. This makes it especially useful in scenarios where experimental validation data are limited. An example of the utility of this approach in a

real-world setting was the study of variants in a honeybee protein.[29] It is important to note, however, that certain limitations persist when applying the IndeLLM Zero-shot scoring methodology. While the proposed approach addresses key challenges, such as eliminating the confounding effects of sequence length differences and mitigating the overemphasis on low-likelihood regions by aggregating probabilities, it does not explicitly incorporate the identities of the inserted or deleted amino acids. As a result, it may misclassify variants in which the specific biochemical properties of the indel residues are the primary drivers of pathogenicity rather than disruptions to local sequence motifs or structural context.

Additionally, the IndeLLM Zero-shot scoring method may underperform on large indels, where substantial portions of the sequence (such as whole domains) are added or removed. In such cases, the model may underestimate the functional impact due to the absence of explicit sequence content, leading to reduced sensitivity in detecting highly disruptive events (see Figure 4). These limitations highlight potential areas for future improvement, such as integrating indel residue identity or adopting hybrid models that combine sequence-based scoring with contextual structural information.

Several avenues remain open for enhancing the model predictive power and interpretability by incorporating additional sources of structural and functional biological information, for example, Pfam domains, CATH functional families, conservation scores, or data on disrupted motifs. Furthermore, the question of how to best leverage more advanced transformer architectures remains an active area of investigation, particularly those that integrate additional data modalities such as structural or functional annotations like ESM3,[16] ProSST,[30] or ProsT5.[17] While current results from the tested models indicate that these multimodal transformer models seem to underperform compared with pure sequence-based PLMs in terms of sequence likelihoods (Table S2), it is important to note that additional data channels other than sequence (for example, structure or functional annotations) were not utilized in those comparisons. As such, significant untapped potential remains for adapting these models to the specific challenges of indel interpretation.

We believe this information can aid in developing the indel predictor field. In line with that goal, much of our attention has been placed on making the method easy to use and accessible. Source code, digital notebooks, and datasets combining data from the most recent published datasets[9,19,23] can be found in the resource availability section.

METHODS

Dataset generation (including handling of long sequences)

We retrieved datasets of indels in the human genome from three recent studies on indel predictions.[9,19,23] The first study, Cannon et al.,[9] evaluated the performance of nine *in silico* pathogenicity prediction tools for small in-frame indels. Their published dataset contains 3,964 human indels (genome build GRCh38) collected from gnomAD, ClinVar, and the Deciphering Developmental Disorders (DDD) study,[31–33] with prediction classifications from nine tools: CADD, CAPICE, FATHMM-indel, MutPred-indel, MutationTaster2021, PROVEAN, SIFT-indel, VEST-indel, and VVP.[8,10,11,34–39] The Fan et al.[23] study presented a PLM-based (ESM1b) pathogenicity predictor for short in-frame indels called SHINE. We retrieved the 1–2 amino acid indel training and validation dataset for SHINE. The Fan et al. dataset contains 5,457 indels from ClinVar and gnomAD. The Brandes et al. study included a dataset that was used to benchmark the performance of a PLM (ESM1b) on in-frame indel predictions, which is part of a genome-wide disease variant effect prediction study.[19] The Brandes et al. dataset contained 3,470 ClinVar indels.

The Cannon et al. dataset did not contain the variant impacts on protein level, so we submitted the reported chromosome position and nucleotide variation to Ensembl Variant Effect Predictor (VEP[40]) to obtain the reference and alternative amino acids for most indels (some transcription IDs were no longer available). Then, we used the available transcription IDs (cross-referenced with the output from VEP) to obtain the full-length peptide sequence from Ensembl BioMart.[41] We quality checked that the dataset contained only in-frame indels, leading to a single protein impact per indel, excluding frameshift variants. The Fan et al. dataset contained protein IDs, which we submitted to BioMart to obtain the full-length peptide sequences (some protein IDs were no longer available). The Brandes et al. dataset included the cropped wild-type and mutated protein sequences that we could use directly in our study.

We combined all the unique indels from the three studies creating our dataset (n = 7,500). The dataset includes 7,500 indels, of which 2,409 are insertions and 5,091 are deletions. Of these, 2,878 are classified as likely pathogenic or pathogenic, while 4,622 are classified as likely benign or benign. The indels range from 1 to 223 amino acids inserted or deleted (length) and represent a distribution of short indels (1–2 amino acids, n = 3,882) and longer indels ($\geq$3 amino acids, n = 3,618) (Figure S1). We used this dataset for all analyses, except the specific performance analysis comparing our scoring approach to the nine indel prediction tools, in which we used the Cannon et al. subset of the dataset. Cannon et al. included the reported scores from the nine prediction tools. The Cannon et al. subset has 3,478 indels from gnomAD (n = 647), ClinVar (n = 2577), and DDD (n = 254), of which 1,115 are insertions and 2,363 are deletions. Of these, 1,518 are classified as likely pathogenic or pathogenic, while 1,960 are classified as likely benign or benign. Based on this specific performance analysis, we found that Provean[37] and MutPred-indel[11] showed no signs of overfitting and were among the few methods that have not been discontinued. We collected prediction scores for all 7,500 indels using Provean and MutPred-indel, which are included in our dataset.

Due to the size limitations of some PLMs, it is not possible to fit sequences with more amino acids than the allowed number of tokens. Models that allow for sequences of any length, such as ESM2, which uses RoPe positional encoding, quickly run into memory limitations due to the memory required to grow quadratically with protein length. Because of this, sequences exceeding 1,000 amino acids were truncated around the indel location, leaving a buffer region of 500 amino acids down and upstream of the indel. Since the attention mechanism grows quadratically with sequence length, most available PLMs have limits on the distance between tokens for which attention is computed, usually capped at 500 amino acids.[15] By cropping the sequences

Table 3. Distribution of indels for the full dataset and splits

Dataset	Total indels	Short deletions	Long deletions	Short insertions	Long insertions	Benign	Pathogenic
Full	7,500	3,374	1,717	1,534	875	4,622	2,878
Training	5,960	2,690	1,357	1,225	688	3,774	2,186
Validation	819	367	194	161	97	453	366
Test	721	317	166	148	90	395	326

and leaving 500 amino acids around the indel location, we guarantee that the maximum amount of the learned self-attention can be used for the effect inference. The resulting cropped sequences have a limit size of 1,000 amino acids plus the length of the indel. In cases where the indel length exceeds the model's size limit, the number of buffer amino acids around the indel is symmetrically reduced to a length that fits the model (1,022 amino acids). All sequences, crops, and annotations used in this study are provided in the resource availability section.

Indel scoring

Brandes et al. described a generalized approach for scoring in-frame indels as a zero-shot inference task.[19] In summary, the described approach calculates a PLL of each sequence s as

$$PLL(s) = \sum_{i=1}^{L} log(P(x_i = s_i|s)),$$

where L is the sequence length, s_i is the amino acid at position i, and $log(P(x_i = s_i|s))$ is the log likelihood predicted by ESM1b for observing the input amino acid s_i at position i given the entire input sequence s. In this context, the output of the PLM is treated as a sequence of random variables $x = x_1, \ldots, x_l$, where each x_i represents the probability distribution for observing 1 of the 20 standard amino acids at position i. The effect score of an in-frame indel is calculated as the difference in PLLs between the mutated (s_{mut}) and the wild-type (s_{WT}) sequences: PLL(s_{mut}) − PLL(s_{WT}). Since the scoring above calculates the PLL of the whole sequence, which was first presented in the Brandes et al. study,[19] we refer to this scoring as "Brandes." One limitation of such a scoring method is that it relies on subtracting incomparable values due to the length difference of the sequences, especially when dealing with longer indels. The study showed attempts to limit that effect using a scaling factor dependent on the length difference but showed no improvements compared with the original Brandes score.[19] In this work, we present an alternative scoring approach to obtain more comparable PLLs by calculating the PLL of only the overlapping amino acids. Since PLMs are context-aware language models, the indels' effect is observable on the marginal probabilities of the rest of the amino acids in the sequence, allowing for the subtraction of comparable PLLs. The scoring can be computed as before, and the PLL of the overlapping regions ($PLL_{overlap}$) can be calculated as

$$PLL_{overlap}(s) = \sum_{i=1}^{L} log(P(x_i = s_i|s \text{ if } s_i \in s_{overlap})),$$

where $s_{overlap}$ is the amino acids present in both the wild-type and the mutated sequences. Building on this rationale, we propose a simplified scoring approach obtained by performing the sum of the probabilities over each of the overlapping positions, decreasing in this way the impact of fluctuations in poorly conserved regions. We refer to this scoring as IndeLLM. The optimal pathogenicity threshold for the entire dataset using the IndeLLM scoring approach is −0.59. This threshold was determined by maximizing the difference between the true- and the false-positive rates (TPRs and FPRs) on the ROC curves.

Finally, we also compare the predictions' performance when using a masking objective for the assessed position or when using inference without masked tokens.

Siamese network architecture

For the Siamese network, we split the dataset into training, validation, and test datasets containing 80%, 10%, and 10% of the indels, respectively. We used CD-HIT[42] to cluster wild-type sequences with a maximum 50% sequence identity (2,902 clusters), and the sequences in each cluster were always kept in the same dataset so that our training, validation, and test datasets contained diverse sequences. The clusters were randomly distributed into the three datasets while ensuring close to equal fractions of indel types, lengths, and pathogenicity classification as in the entire dataset. We considered indels of 1–2 amino acid short, while the remaining were long. See Table 3 for the distributions per dataset.

To enhance indel classification and explore transfer learning potential using PLMs, we implemented a shallow feedforward neural network (FNN) inspired by the architecture described in VariPred for SNVs.[20] The FNN was trained solely on the class labels, keeping the PLM parameters frozen. A simple grid search was employed for hyperparameter optimization, with the final parameters for each model detailed in Figure 2. To mitigate overfitting, the FNN was designed with a single fully connected hidden layer. Each model was trained five times with different initial weights to evaluate reliability.

The input features extracted for the models were as follows: mean embeddings of the last hidden layer for the wild-type sequence, mean embeddings of the last hidden layer for the mutant sequence, mean embeddings of the last hidden layer for the deleted or inserted amino acids, the IndeLLM score, the length of the indel, and the type of indel (0 for deletion, 1 for insertion). For models utilizing indel embeddings, the embeddings corresponding to the indel region were excluded from the longest sequence's embeddings to ensure that only corresponding regions of the protein were compared.

Four final model architectures were developed, each leveraging a different combination of these features.

(1) Model 1: a basic Siamese network that used the mean embeddings from the last hidden layer of the PLM for the whole wild-type and mutant sequences.

CellPress
OPEN ACCESS

(2) Model 2: built on model 1 by adding the IndeLLM score as an additional input parameter.
(3) Model 3: extended model 2 by incorporating the indel type (insertion or deletion) and indel length as additional parameters.
(4) Model 4: this model took a more advanced approach by splitting embeddings into overlapping and non-overlapping regions. The inputs included the mean embeddings from the overlapping regions of the wild-type and mutant sequences, the mean embeddings of the indel (non-overlapping region), the indel type, and its length. This biologically informed architecture aimed to capture sequence structure better.

Each model was evaluated to determine the contribution of its specific features to the indel classification task. The sequence embeddings were extracted using ESM2 with 650M parameters. A final output layer with two nodes and a SoftMax activation function was used to classify variants as pathogenic or benign. Only the pathogenic node's output was considered for classification. The AUC on the validation dataset was used as the early stopping criterion during training. The best-performing pathogenicity threshold for all the data is 0.46.

Performance analysis

To compare the performance of the pathogenicity prediction tools and PLMs, we computed the AUC of the ROC, the F1 score, and the MCC score and confusion matrices using Scikit-learn.[43] The ROC curve provided the FPRs and TPRs at different threshold values. When comparing any approach in our study, we used the calculated FPR and TPR to find the optimal threshold, rather than the default threshold provided by each pathogenicity prediction tool, to make a fair comparison. The FPR and TPR were also used to calculate the ROC curve's AUC. Our calculations are provided in our analysis Jupyter Notebooks.

Interpretability analysis

Since PLMs are context aware, one can compare the changes of the predicted amino acid probabilities at each position due to the surrounding context by subtracting the individual probabilities from each other for the amino acids that are overlapping:

$$P(x_i = s_i|s_{mut}) - P(x_i = s_i|s_{wt}),$$

where $(s_i|s_{\mathrm{mut}})$is the first amino acid in the mutated sequence s_{mut}, and $(s_i|s_{\mathrm{wt}})$ is the same amino acid but in the wild-type sequence s_{wt}. This results in a different score for each position in the sequence.

These localized changes in probabilities can then be mapped back to the 3D structure of the protein of interest. This allows one to observe the regions where the model predicts significant changes due to the indel and understand which positions drive the pathogenicity prediction results.

To illustrate this approach, we identified genes in our dataset with multiple indels reported, both pathogenic and benign. We selected two proteins, FGFR1 and GLMN, which had their 3D protein structure resolved at high resolution. We show two domains of FGFR1, the apo structure of the intracellular tyrosine kinase domain (PDB: 4UWY,[44] Figure 5E) and the first of the three extracellular Ig-like domains (PDB: 2CR3,[45] Figure 5F), and the complete GLMN chain extracted from the GLMN-RBX1-CUL1 complex (PDB: 4F52,[28] Figure 5I). The probability changes (difference scores) were obtained by aligning the wild-type and mutated sequences using the Bio.AlignIO package from Biopython[46] and color was rendered on the structure using PyMOL.

RESOURCE AVAILABILITY

Lead contact

Requests for further information and resources should be directed to and will be fulfilled by the lead contact, Oriol Gracia Carmona (o.carmona@ucl.ac.uk).

Materials availability

This study did not generate new unique reagents or materials.

Data and code availability

- All the data used on this project can be found on GitHub (https://github.com/OriolGraCar/IndeLLM) and HuggingFace.[47]
- The source code for IndeLLM, all analyses, and examples are available in our GitHub repository (https://github.com/OriolGraCar/IndeLLM). The original code has also been deposited at Zenodo[48] and is publicly available) as of the date of publication. We also released a plug-and-play Google Colab version of the code,[48] which is also available at https://colab.research.google.com/github/OriolGraCar/IndeLLM/blob/main/IndeLLM.ipynb.

ACKNOWLEDGMENTS

The authors acknowledge support from the Biotechnology and Biological Sciences Research Council (BB/T002212/1 to F.F. as principal investigator). F.F. and O.G.C. are thankful for the BHF grant (RG/F/22/110079). V.L. and G.V.A. acknowledge The Research Council of Norway grant nos. 335244 and 350231 for funding toward running costs, travel grants, and conference support.

AUTHOR CONTRIBUTIONS

O.G.C. and V.L. contributed to the conceptualization, visualization, methodology, software development, validation, formal analysis, data curation, writing of the original draft, and review and editing of the manuscript. F.F., C.O., and G.V.A. contributed to the writing of the original draft as well as to the review and editing of the manuscript.

DECLARATION OF INTERESTS

The authors declare no competing interests.

DECLARATION OF GENERATIVE AI AND AI-ASSISTED TECHNOLOGIES IN THE WRITING PROCESS

During the preparation of this work, the authors used generative-AI-powered tools in order to fix grammar and spelling mistakes. The authors have reviewed and edited the content as needed and take full responsibility for the content of the publication.

SUPPLEMENTAL INFORMATION

Supplemental information can be found online at https://doi.org/10.1016/j.patter.2025.101425.

Received: June 11, 2025
Revised: August 20, 2025
Accepted: October 21, 2025
Published: November 26, 2025

REFERENCES

1. Mullaney, J.M., Mills, R.E., Pittard, W.S., and Devine, S.E. (2010). Small insertions and deletions (INDELs) in human genomes. Hum. Mol. Genet. *19*, R131–R136. https://doi.org/10.1093/hmg/ddq400.
2. Mills, R.E., Luttig, C.T., Larkins, C.E., Beauchamp, A., Tsui, C., Pittard, W.S., and Devine, S.E. (2006). An initial map of insertion and deletion (INDEL) variation in the human genome. Genome Res. *16*, 1182–1190. https://doi.org/10.1101/gr.4565806.
3. Kim, R., and Guo, J.t. (2010). Systematic analysis of short internal indels and their impact on protein folding. BMC Struct. Biol. *10*, 24. https://doi.org/10.1186/1472-6807-10-24.
4. Lin, M., Whitmire, S., Chen, J., Farrel, A., Shi, X., and Guo, J.T. (2017). Effects of short indels on protein structure and function in human genomes. Sci. Rep. *7*, 9313. https://doi.org/10.1038/s41598-017-09287-x.
5. Farinha, C.M., and Callebaut, I. (2022). Molecular mechanisms of cystic fibrosis – how mutations lead to misfunction and guide therapy. Biosci. Rep. *42*, BSR20212006. https://doi.org/10.1042/BSR20212006.
6. Sergouniotis, P.I., Barton, S.J., Waller, S., Perveen, R., Ellingford, J.M., Campbell, C., Hall, G., Gillespie, R.L., Bhaskar, S.S., Ramsden, S.C., et al. (2016). The role of small in-frame insertions/deletions in inherited eye disorders and how structural modelling can help estimate their pathogenicity. Orphanet J. Rare Dis. *11*, 125. https://doi.org/10.1186/s13023-016-0505-0.
7. Ye, K., Wang, J., Jayasinghe, R., Lameijer, E.-W., McMichael, J.F., Ning, J., McLellan, M.D., Xie, M., Cao, S., Yellapantula, V., et al. (2016). Systematic discovery of complex insertions and deletions in human cancers. Nat. Med. *22*, 97–104. https://doi.org/10.1038/nm.4002.
8. Douville, C., Masica, D.L., Stenson, P.D., Cooper, D.N., Gygax, D.M., Kim, R., Ryan, M., and Karchin, R. (2016). Assessing the Pathogenicity of Insertion and Deletion Variants with the Variant Effect Scoring Tool (VEST-Indel). Hum. Mutat. *37*, 28–35. https://doi.org/10.1002/humu.22911.
9. Cannon, S., Williams, M., Gunning, A.C., and Wright, C.F. (2023). Evaluation of in silico pathogenicity prediction tools for the classification of small in-frame indels. BMC Med. Genomics *16*, 36. https://doi.org/10.1186/s12920-023-01454-6.
10. Steinhaus, R., Proft, S., Schuelke, M., Cooper, D.N., Schwarz, J.M., and Seelow, D. (2021). MutationTaster2021. Nucleic Acids Res. *49*, W446–W451. https://doi.org/10.1093/nar/gkab266.
11. Pagel, K.A., Antaki, D., Lian, A., Mort, M., Cooper, D.N., Sebat, J., Iakoucheva, L.M., Mooney, S.D., and Radivojac, P. (2019). Pathogenicity and functional impact of non-frameshifting insertion/deletion variation in the human genome. PLoS Comput. Biol. *15*, e1007112. https://doi.org/10.1371/journal.pcbi.1007112.
12. Agarwal, A., Zhao, F., Jiang, Y., and Chen, L. (2023). TIVAN-indel: a computational framework for annotating and predicting non-coding regulatory small insertions and deletions. Bioinformatics *39*, btad060. https://doi.org/10.1093/bioinformatics/btad060.
13. Meier, J., Rao, R., Verkuil, R., Liu, J., Sercu, T., and Rives, A. (2021). Language models enable zero-shot prediction of the effects of mutations on protein function. Preprint at bioRxiv. https://doi.org/10.1101/2021.07.09.450648.
14. Rives, A., Meier, J., Sercu, T., Goyal, S., Lin, Z., Liu, J., Guo, D., Ott, M., Zitnick, C.L., Ma, J., and Fergus, R. (2021). Biological structure and function emerge from scaling unsupervised learning to 250 million protein sequences. Proc. Natl. Acad. Sci. *118*, e2016239118. https://doi.org/10.1073/pnas.2016239118.
15. Lin, Z., Akin, H., Rao, R., Hie, B., Zhu, Z., Lu, W., Smetanin, N., Verkuil, R., Kabeli, O., Shmueli, Y., et al. (2023). Evolutionary-scale prediction of atomic-level protein structure with a language model. Science *379*, 1123–1130. https://doi.org/10.1126/science.ade2574.
16. Hayes, T., Rao, R., Akin, H., Sofroniew, N.J., Oktay, D., Lin, Z., Verkuil, R., Tran, V.Q., Deaton, J., Wiggert, M., et al. (2024). Simulating 500 million years of evolution with a language model. Preprint at bioRxiv. https://doi.org/10.1101/2024.07.01.600583.
17. Heinzinger, M., Weissenow, K., Sanchez, J.G., Henkel, A., Mirdita, M., Steinegger, M., and Rost, B. (2024). Bilingual language model for protein sequence and structure. NAR Genom. Bioinform. *6*, lqae150. https://doi.org/10.1093/nargab/lqae150.
18. Ruffolo, J.A., Gray, J.J., and Sulam, J. (2021). Deciphering antibody affinity maturation with language models and weakly supervised learning. Preprint at arXiv. https://doi.org/10.48550/arXiv.2112.07782.
19. Brandes, N., Goldman, G., Wang, C.H., Ye, C.J., and Ntranos, V. (2023). Genome-wide prediction of disease variant effects with a deep protein language model. Nat. Genet. *55*, 1512–1522. https://doi.org/10.1038/s41588-023-01465-0.
20. Lin, W., Wells, J., Wang, Z., Orengo, C., and Martin, A.C.R. (2024). Enhancing missense variant pathogenicity prediction with protein language models using VariPred. Sci. Rep. *14*, 8136. https://doi.org/10.1038/s41598-024-51489-7.
21. Lampert, C.H., Nickisch, H., and Harmeling, S. (2009). Learning to detect unseen object classes by between-class attribute transfer. In 2009 IEEE Conference on Computer Vision and Pattern Recognition, pp. 951–958. https://doi.org/10.1109/CVPR.2009.5206594.
22. Notin, P., Kollasch, A., Ritter, D., van Niekerk, L., Paul, S., Spinner, H., Rollins, N., Shaw, A., Orenbuch, R., Weitzman, R., et al. (2023). ProteinGym: Large-Scale Benchmarks for Protein Fitness Prediction and Design. Adv. Neural Inf. Process. Syst. *36*, 64331–64379.
23. Fan, X., Pan, H., Tian, A., Chung, W.K., and Shen, Y. (2023). SHINE: protein language model-based pathogenicity prediction for short inframe insertion and deletion variants. Brief. Bioinform. *24*, bbac584. https://doi.org/10.1093/bib/bbac584.
24. Nori, D., Singireddy, S., and Have, M.T. (2023). Identification of Knowledge Neurons in Protein Language Models. Preprint at arXiv. https://doi.org/10.48550/arXiv.2312.10770.
25. Tsui, D., and Aghazadeh, A. (2024). On Recovering Higher-order Interactions from Protein Language Models. Preprint at arXiv. https://doi.org/10.48550/arXiv.2405.06645.
26. Dai, S., Zhou, Z., Chen, Z., Xu, G., and Chen, Y. (2019). Fibroblast Growth Factor Receptors (FGFRs): Structures and Small Molecule Inhibitors. Cells *8*, 614. https://doi.org/10.3390/cells8060614.
27. Mirdita, M., Schütze, K., Moriwaki, Y., Heo, L., Ovchinnikov, S., and Steinegger, M. (2022). ColabFold: making protein folding accessible to all. Nat. Methods *19*, 679–682. https://doi.org/10.1038/s41592-022-01488-1.
28. Duda, D.M., Olszewski, J.L., Tron, A.E., Hammel, M., Lambert, L.J., Waddell, M.B., Mittag, T., DeCaprio, J.A., and Schulman, B.A. (2012). Structure of a Glomulin-RBX1-CUL1 Complex: Inhibition of a RING E3 Ligase through Masking of Its E2-Binding Surface. Mol. Cell *47*, 371–382. https://doi.org/10.1016/j.molcel.2012.05.044.
29. Leipart, V., Gracia Carmona, O., Orengo, C., Fraternali, F., and Amdam, G.V. (2025). Assessing structure-function impacts on Vitellogenin by leveraging allelic variant occurring in honey bee subspecies Apis mellifera mellifera. iScience *28*, 113241. https://doi.org/10.1016/j.isci.2025.113241.
30. Li, M., Tan, P., Ma, X., Zhong, B., Yu, H., Zhou, Z., Ouyang, W., Zhou, B., Hong, L., and Tan, Y. (2024). ProSST: Protein Language Modeling with Quantized Structure and Disentangled Attention. Preprint at bioRxiv. https://doi.org/10.1101/2024.04.15.589672.
31. Karczewski, K.J., Francioli, L.C., Tiao, G., Cummings, B.B., Alföldi, J., Wang, Q., Collins, R.L., Laricchia, K.M., Ganna, A., Birnbaum, D.P., et al. (2020). The mutational constraint spectrum quantified from variation in 141,456 humans. Nature *581*, 434–443. https://doi.org/10.1038/s41586-020-2308-7.
32. Landrum, M.J., Lee, J.M., Riley, G.R., Jang, W., Rubinstein, W.S., Church, D.M., and Maglott, D.R. (2014). ClinVar: public archive of relationships among sequence variation and human phenotype. Nucleic Acids Res. *42*, D980–D985. https://doi.org/10.1093/nar/gkt1113.

CellPress
OPEN ACCESS

33. Wright, C.F., Fitzgerald, T.W., Jones, W.D., Clayton, S., McRae, J.F., van Kogelenberg, M., King, D.A., Ambridge, K., Barrett, D.M., Bayzetinova, T., et al. (2015). Genetic diagnosis of developmental disorders in the DDD study: a scalable analysis of genome-wide research data. Lancet *385*, 1305–1314. https://doi.org/10.1016/S0140-6736(14)61705-0.

34. Rentzsch, P., Schubach, M., Shendure, J., and Kircher, M. (2021). CADD-Splice—improving genome-wide variant effect prediction using deep learning-derived splice scores. Genome Med. *13*, 31. https://doi.org/10.1186/s13073-021-00835-9.

35. Li, S., van der Velde, K.J., de Ridder, D., van Dijk, A.D.J., Soudis, D., Zwerwer, L.R., Deelen, P., Hendriksen, D., Charbon, B., van Gijn, M.E., et al. (2020). CAPICE: a computational method for Consequence-Agnostic Pathogenicity Interpretation of Clinical Exome variations. Genome Med. *12*, 75. https://doi.org/10.1186/s13073-020-00775-w.

36. Ferlaino, M., Rogers, M.F., Shihab, H.A., Mort, M., Cooper, D.N., Gaunt, T.R., and Campbell, C. (2017). An integrative approach to predicting the functional effects of small indels in non-coding regions of the human genome. BMC Bioinf. *18*, 442. https://doi.org/10.1186/s12859-017-1862-y.

37. Choi, Y., Sims, G.E., Murphy, S., Miller, J.R., and Chan, A.P. (2012). Predicting the Functional Effect of Amino Acid Substitutions and Indels. PLoS One *7*, e46688. https://doi.org/10.1371/journal.pone.0046688.

38. Hu, J., and Ng, P.C. (2013). SIFT Indel: Predictions for the Functional Effects of Amino Acid Insertions/Deletions in Proteins. PLoS One *8*, e77940. https://doi.org/10.1371/journal.pone.0077940.

39. Flygare, S., Hernandez, E.J., Phan, L., Moore, B., Li, M., Fejes, A., Hu, H., Eilbeck, K., Huff, C., Jorde, L., et al. (2018). The VAAST Variant Prioritizer (VVP): ultrafast, easy to use whole genome variant prioritization tool. BMC Bioinf. *19*, 57. https://doi.org/10.1186/s12859-018-2056-y.

40. McLaren, W., Gil, L., Hunt, S.E., Riat, H.S., Ritchie, G.R.S., Thormann, A., Flicek, P., and Cunningham, F. (2016). The Ensembl Variant Effect Predictor. Genome Biol. *17*, 122. https://doi.org/10.1186/s13059-016-0974-4.

41. Kinsella, R.J., Kähäri, A., Haider, S., Zamora, J., Proctor, G., Spudich, G., Almeida-King, J., Staines, D., Derwent, P., Kerhornou, A., et al. (2011). Ensembl BioMarts: a hub for data retrieval across taxonomic space. Database J. Biol. Databases Curation *2011*, bar030. https://doi.org/10.1093/database/bar030.

42. Fu, L., Niu, B., Zhu, Z., Wu, S., and Li, W. (2012). CD-HIT: accelerated for clustering the next-generation sequencing data. Bioinformatics *28*, 3150–3152. https://doi.org/10.1093/bioinformatics/bts565.

43. Pedregosa, F., Varoquaux, G., Gramfort, A., Michel, V., Thirion, B., Grisel, O., Blondel, M., Prettenhofer, P., Weiss, R., Dubourg, V., et al. (2011). Scikit-learn: Machine Learning in Python. J. Mach. Learn. Res. *12*, 2825–2830.

44. Bunney, T.D., Wan, S., Thiyagarajan, N., Sutto, L., Williams, S.V., Ashford, P., Koss, H., Knowles, M.A., Gervasio, F.L., Coveney, P.V., and Katan, M. (2015). The Effect of Mutations on Drug Sensitivity and Kinase Activity of Fibroblast Growth Factor Receptors: A Combined Experimental and Theoretical Study. EBioMedicine *2*, 194–204. https://doi.org/10.1016/j.ebiom.2015.02.009.

45. Hatta, R., Hayashi, F., Yoshida, M., and Yokoyama, S.; RIKEN Structural Genomics/Proteomics Initiative (RSGI) (2005). RCSB PDB - 2CR3: Solution structure of the first Ig-like domain of human fibroblast growth factor receptor 1. https://www.rcsb.org/structure/2CR3.

46. Cock, P.J.A., Antao, T., Chang, J.T., Chapman, B.A., Cox, C.J., Dalke, A., Friedberg, I., Hamelryck, T., Kauff, F., Wilczynski, B., and de Hoon, M.J.L. (2009). Biopython: freely available Python tools for computational molecular biology and bioinformatics. Bioinformatics *25*, 1422–1423. https://doi.org/10.1093/bioinformatics/btp163.

47. Gracia Carmona, O., Leipart, V., Amdam, G.V., Orengo, C., and Fraternali, F. (2025). Leveraging protein language models and a scoring function for indel characterisation and transfer learning. HuggingFace. https://doi.org/10.57967/hf/6581.

48. Gracia Carmona, O., Leipart, V., Amdam, G.V., Orengo, C., and Fraternali, F. (2025). Leveraging protein language models and a scoring function for indel characterisation and transfer learning. Zenodo. https://doi.org/10.5281/zenodo.17196223.

Patterns, Volume *7*

Supplemental information

Leveraging protein language models and a scoring function for indel characterization and transfer learning

Oriol Gracia Carmona, Vilde Leipart, Gro V. Amdam, Christine Orengo, and Franca Fraternali

Supplemental Notes

S1. Dataset curation

In this work, we publish a dataset of 7500 indels. These indels are combined from three studies (Cannon et al. [1], Fan et al. [2] and Brandes et al. [3]). IndeLLM requires protein sequences as input. Using gene and transcriptions IDs, we collected the protein sequences for the indels where this information was not included. The curation of all indels (ensuring correct protein sequence, indel annotation, excluding any frameshift or delins (which are deletions followed by insertions resulting in identical length of wildtype and mutated sequence)) resulted in the exclusion of hundreds of indels. However, the final dataset of 7500 indels is thoroughly curated and represents, to our knowledge, the largest dataset of in-frame indels with pathogenicity classifications. The dataset includes 2409 insertions and 5091 deletions. Of these, 2878 are classified as likely pathogenic or pathogenic, while 4622 are classified as likely benign or benign. The indels range from 1 to 223 in length and represent a distribution of short indels (1-2 amino acid, n = 3882) and longer indels (>= 3 amino acids, n = 3618). See Figure S1 for distribution.

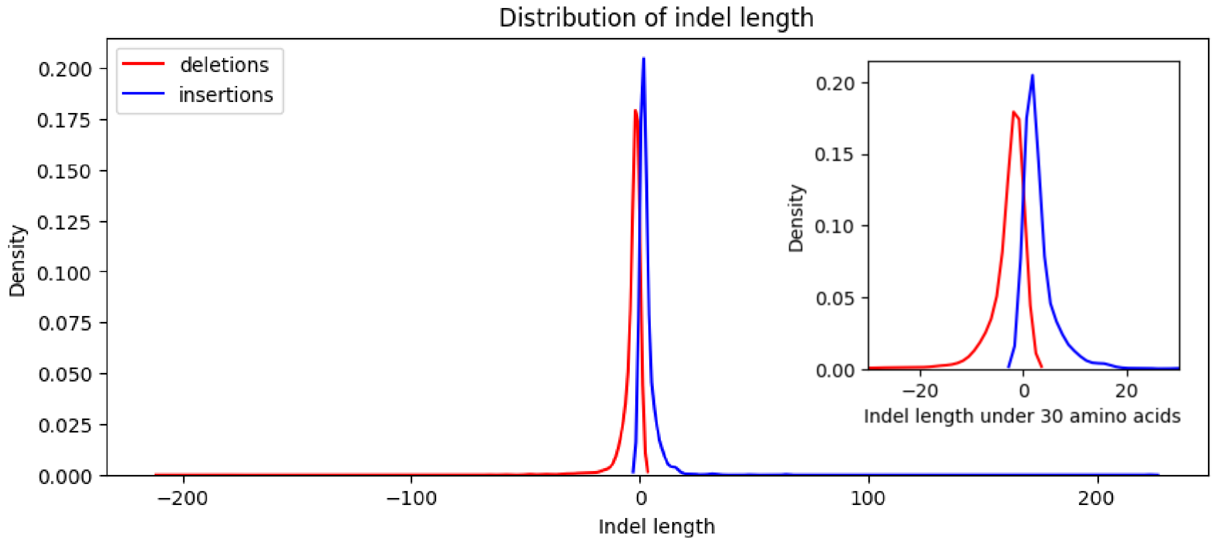

Figure S1 Distribution of indel length: The distribution of indels by length (deletions in red and insertions in blue).

S2. Scoring IndeLLM Zero-shot probabilities vs log-probabilities

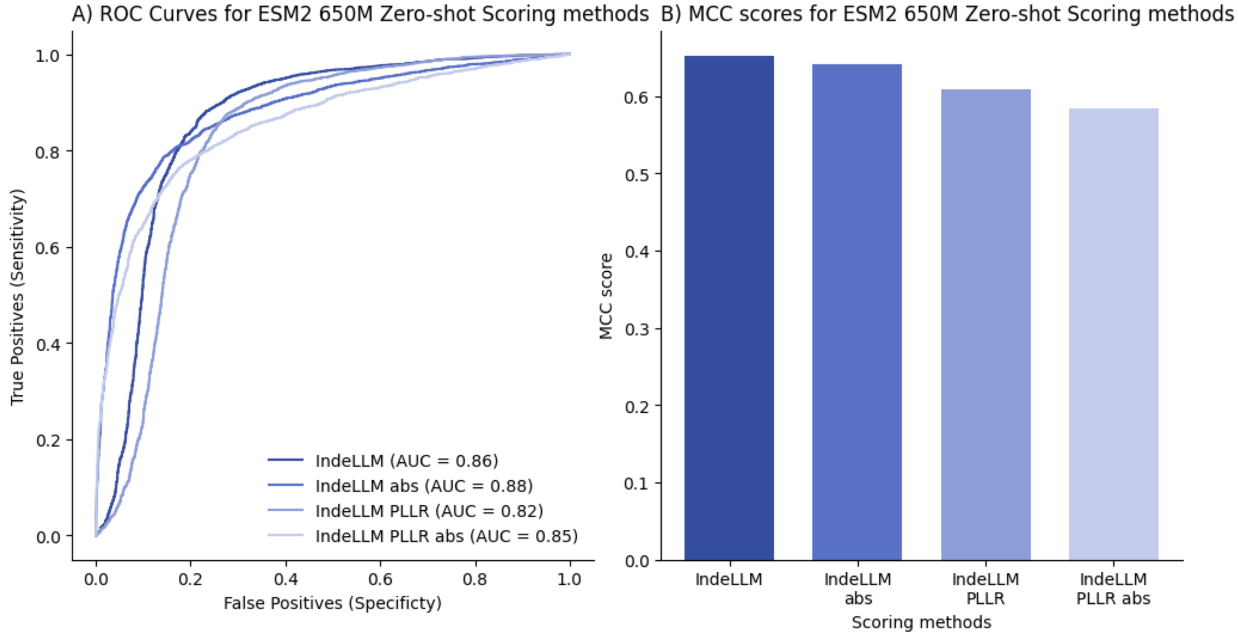

Figure S2 Zero-shot performance evaluation: Evaluation of different Zero-shot scoring methods using the sum of probabilities and the sum of log-probabilities (PLLR) of ESM2 (650M parameters) to calculate the IndeLLM score. The different scoring methods were IndeLLM (sum probabilities of overlapping regions), IndeLLM abs (using the absolute probability of overlapping regions), IndeLLM PLLR (sum of PLLR), and IndeLLM PLLR abs (using the absolute PLLR of overlapping regions). We compared the scoring methods by A) plotting the ROC curve and calculating the AUC and B) calculating the MCC scores. See Table S1 for all scores.

Full dataset (n = 7500)

PLMs*	MCC	F1	AUC
IndeLLM	0.65	0.87	0.86
IndeLLM abs	0.64	0.86	0.88
IndeLLM PLLR	0.61	0.85	0.82
IndeLLM PLLR abs	0.58	0.84	0.85

*Table S1 Zero-shot performance evaluation: The calculated MCC, F1 and AUC scores for the additional IndeLLM scores (*Scoring methods results reported are calculated using the probabilities from ESM2 (650M parameters)).*

S3. Comparing Zero-shot for 7 PLMs

We have studied the performance and transferability of 7 PLMs, ESM1v [4], ESM1b [5], ProtTrans [6], ESM2 at three different sizes (150M, 650M and 3B parameters) [7] and ESM3 (1.4B parameters) [8]. All PLMs were run using the HuggingFace transformers library and the available pre-trained parameters from HuggingFace, except ESM3, which was run using the code available on the official GitHub repository of the Model (https://github.com/evolutionaryscale/esm). ESM3 is a multimodal generative language model that can be inputted with information for different tracks. This work's goal was to examine the performance of language models using protein sequence alone, so only the protein track was used to assess the performance of ESM3.

Using the 7500 indels dataset and our IndeLLM score, we found that three PLMs performed the best (ESM1b, ESM2-650M, and ESM2-3B), with similar accuracies: MCC of 0.68 vs. 0.65 vs. 0.64, respectively (Figure S3 and Table S2). Due to the flexibility of rotatory positional embedding, ESM2-650M is the best candidate for encoding longer sequences without extra preprocessing steps.

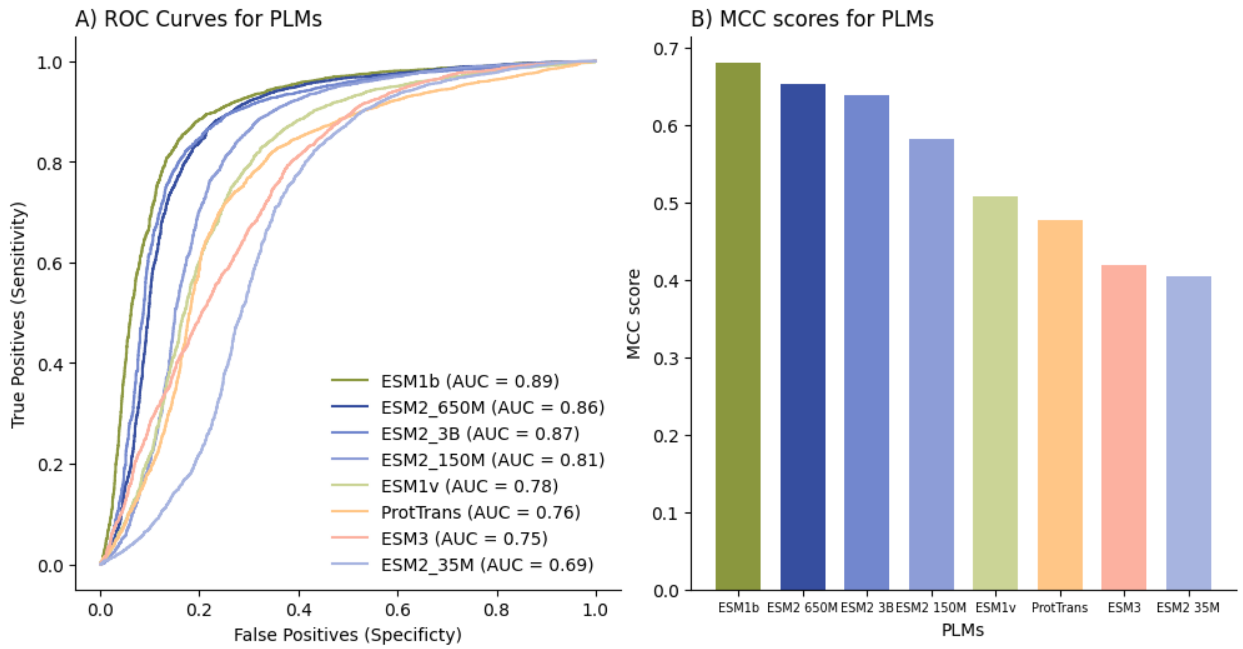

Figure S3 PLMs performance on indels: Evaluation of different PLMs using the local scoring approach. The different PLMs are ESM1 (b and v, green), ESM2 (35M, 150M, 650M and 3B parameters, blue), ESM3 (orange) and ProtTrans (red). We compared the scoring methods by A) plotting the ROC curve and calculating the AUC and B) calculating the MCC scores.

Full dataset (n = 7500)

PLMs*	MCC	F1	AUC
ESM1b	0.68	0.87	0.89
ESM2 (650M parameters)	0.65	0.87	0.86
ESM2 (3B parameters)	0.64	0.85	0.87
ESM2 (150M parameters)	0.58	0.85	0.81
ESM1v	0.51	0.82	0.78
ProtTrans	0.48	0.80	0.76
ESM3	0.42	0.78	0.75
ESM2 (35M parameters)	0.40	0.78	0.69

*Table S2 PLMs performance on indels: The calculated MCC, F1 and AUC scores for the 7 PLMs (*All results use indeLLM score).*

Models with additional parameters, such as ESM2-3B (MCC score 0.64), did not perform better than the smaller models, suggesting that these smaller checkpoints may be more suitable to represent indels. The trend of bigger models not always correlating with better performance has also been previously observed in the pure Large Language Models (LLMs) [9]. ESM3 performed worst among the tested ESM models (MCC 0.42). However, it is important to note that we only used the sequence track in our analysis, which leaves the question of how ESM3 would perform if extra features were provided in the other tracks. We also evaluated additional encoder–decoder architectures, specifically ProT5 and ProsT5 [10]. Given the design of these models, likelihoods were derived from their decoder layers. For ProsT5, sequences were first converted into 3DI representations, which were then reprocessed by the model to extract amino acid likelihoods. In contrast, ProT5 was queried in an autoregressive fashion. Both models exhibited poor performance in terms of likelihood estimation, yielding an AUC < 0.52. Nonetheless, this limitation does not preclude their potential utility for indel representation, as their embeddings may still encode relevant information for downstream applications. Further investigation into how these models' embeddings can be leveraged for structural, functional, or evolutionary analyses remains a promising avenue for future work.

S4. Comparing Brandes and IndeLLM scores to nine prediction tools

Cannon et al. 2023 [1] include pathogenicity prediction from nine tools: CADD, CAPICE, FATHMM-indel, MutPred-Indel, MutationTaster2021, PROVEAN, SIFT-indel, VEST-indel, and VVP. We used the Cannon et al. subset in the full dataset, which holds 3478 indels from gnomAD (n = 647), ClinVar (n = 2577), and DDD (n = 254), where 1115 are insertions and 2363 are deletions. Of these, 1518 are classified as likely pathogenic or pathogenic, while 1960 are classified as likely benign or benign. We compared performance using the Brandes and IndeLLM score to the nine indels predictors reported (Figure S4 and Table S3). Brandes score underperformed, ranking last among the tested methods (MCC score: 0.38), while using the IndeLLM score resulted in a performance with similar accuracy to the best-performing predictors (MCC scores: IndeLLM = 0.69, MutationTaster2021 = 0.93, PROVEAN = 0.77 and Vest-indel = 0.74).

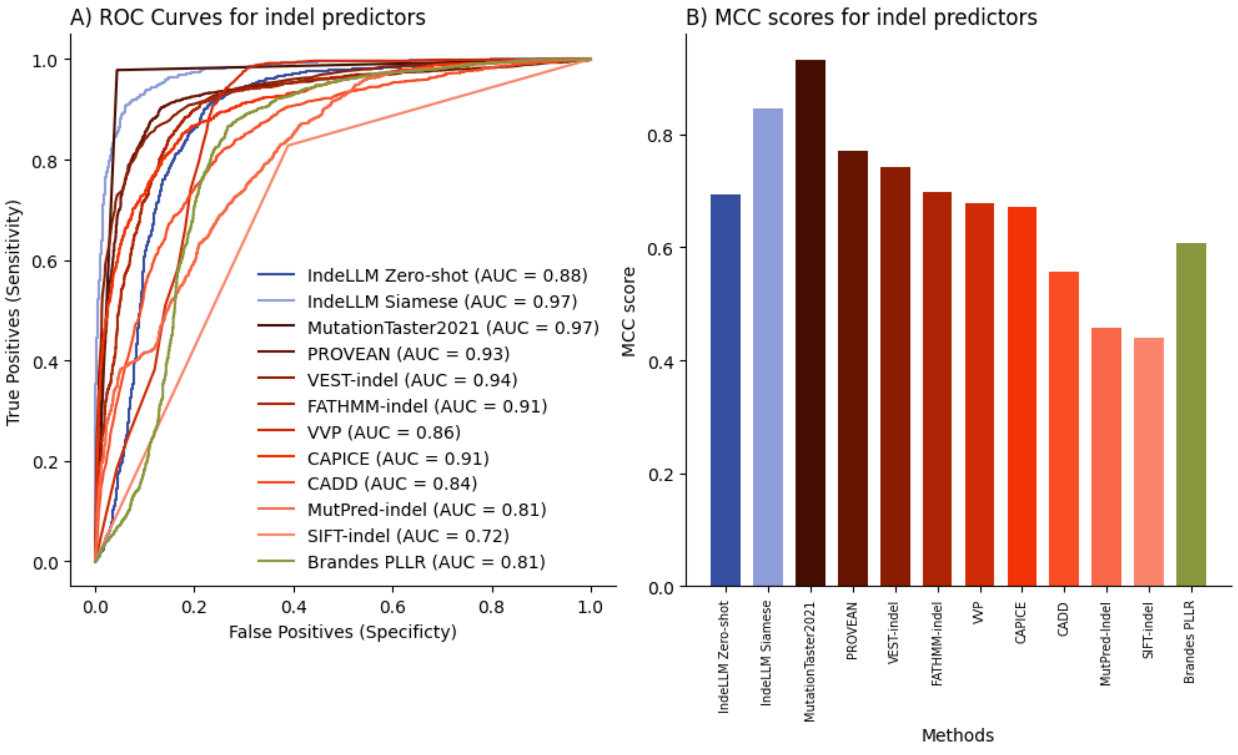

Figure S4 Comparison of predictive methods: Evaluation of different prediction tools (red) to IndeLLM score (dark blue), IndeLLM siamese (dark blue) and Brandes Score (green). We compared the scoring methods by A) plotting the ROC curve and calculating the AUC and B) calculating the MCC scores.

Cannon et al. dataset (n = 3478)

**Published indel predictors**	**MCC**	**F1**	**AUC**
MutationTaster2021	0.93	0.97	0.97
PROVEAN	0.77	0.90	0.93
VEST-indel	0.74	0.89	0.94
FATHMM-indel	0.70	0.86	0.91
VVP	0.68	0.83	0.86
CAPICE	0.67	0.85	0.91
CADD	0.56	0.79	0.84
MutPred-indel	0.46	0.72	0.81
SIFT-indel	0.44	0.70	0.72
**Scoring method***			
IndeLLM Zero-shot	0.69	0.87	0.88
Brandes PLLR	0.61	0.84	0.81
**Supervised**			
IndeLLM Siamese	0.85	0.91	0.97

*Table S3 Comparison of predictive methods: The calculated MCC, F1 and AUC scores for the prediction tools and IndeLLM zero-shot, Brandes scores (*Scoring methods results reported are calculated using the probabilities from ESM2 (650M parameters)), and the supervised model IndeLLM siamese.*

S5. Evaluating the level of overfitting

Cannon et al. suggested using the DDD indels (n=254) [1] as a good candidate to examine overfitting on different methods with labeled data since the dataset was made available after those methods were published. We expanded the original analysis of overfitting to all the reported methods (Figure S5 and Table S4). Several methods displayed degrees of overfitting. Among them was the best-performing method, MutationTaster2021, having its performance cut from MCC 0.95 to 0.51 (Fig S5E and S5L). No overfitting was observed for PROVEAN and MutPred-Indel (MCC dropped 0.11 and 0.10, respectively, Fig S5D, S5F, and S5L). As expected, the performance of IndeLLM and Brandes showcased no overfitting since no indel sequences were used at any point to train or readjust any of the original weights (Fig S5J, S5K and S5L).

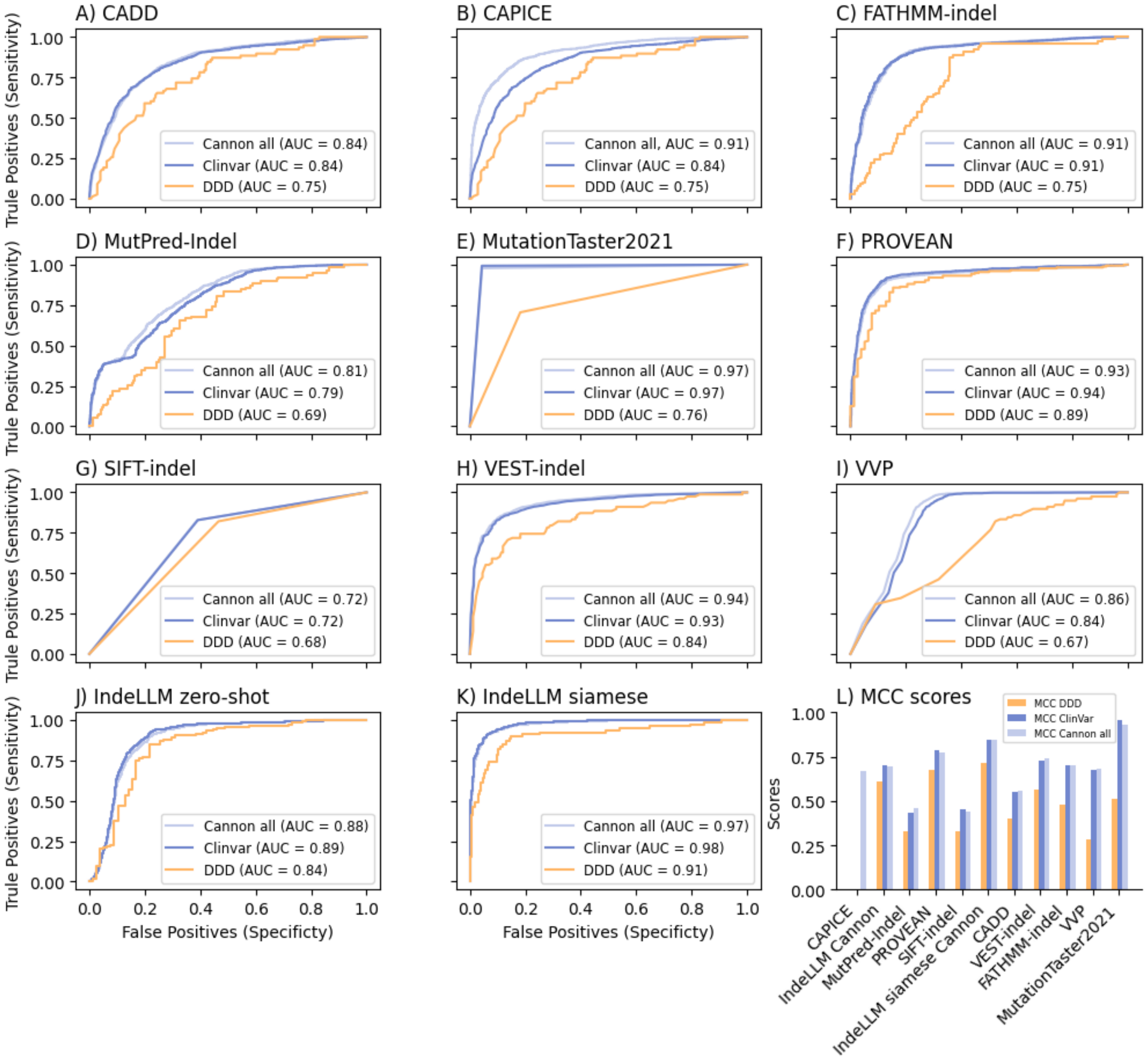

Figure S5 Performances across datasets of predictive methods: Evaluation of the level of

overfitting of pathogenicity prediction tools. Panel A-I shows the AUC for nine prediction tools. Panel J shows the AUC for IndeLLM scoring. Panel K shows the AUC for IndeLLM siamese. We calculate AUC using all Cannon et al. indels (light blue), the Cannon et al. indels collected from ClinVar (dark blue) and the Cannon et al. indels collected from DDD (orange). In panel L, we show the calculated MCC scores for each method per dataset split and sorted by the difference between MCC ClinVar and MCC DDD. CAPICE has no calculations of MCC for the ClinVar and DDD data splits since the tool predicts all indels as either pathogenic or benign, making the binary prediction one-dimensional.

Cannon et al. Dataset split by indel origin, ClinVar (n = 2577) and DDD (n = 254)

	**ClinVar**			**DDD**			**DDD - ClinVar**
**Published indel predictors**	**MCC**	**F1**	**AUC**	**MCC**	**F1**	**AUC**	**MCC diff**
MutPred-Indel	0.43	0.65	0.79	0.33	0.65	0.69	0.10
PROVEAN	0.78	0.88	0.94	0.68	0.89	0.89	0.11
SIFT-indel	0.45	0.67	0.72	0.33	0.66	0.68	0.12
CADD	0.55	0.75	0.84	0.40	0.69	0.75	0.15
VEST-indel	0.73	0.85	0.93	0.57	0.87	0.84	0.16
FATHMM-indel	0.70	0.83	0.91	0.48	0.75	0.75	0.22
VVP	0.67	0.79	0.84	0.28	0.61	0.67	0.39
MutationTaster2021	0.95	0.97	0.97	0.51	0.84	0.76	0.45
CAPICE	0**	0.61	0.84	0**	0.82	0.75	0.67
**IndeLLM**	**MCC**	**F1**	**AUC**	**MCC**	**F1**	**AUC**	**MCC diff**
IndeLLM zero-shot	0.70	0.84	0.89	0.61	0.87	0.84	0.09
IndeLLM Siamese	0.84	0.93	0.98	0.71	0.80	0.91	0.13

*Table S4 Performances across datasets of predictive methods: The calculated MCC, F1 and AUC scores for the prediction tools and the two IndeLLM methods for the ClinVar and DDD data splits. The final column calculates the difference in MCC (*Scores are calculated using the probabilities from ESM2 (650M parameters), **MCC not calculated due to CAPICE binary predictions being one-dimensional).*

To evaluate our supervised model, IndeLLM siamese, we used a never-seen-before indels dataset from ProteinGym [11]. We ensured that the dataset only had unique indels by aligning the mutated protein sequences from ProteinGym dataset (n=1695) to all mutated protein sequences in our indels dataset (n=7500). The dataset from ProteinGym is named “holdout dataset” and includes 807 insertions and 888 deletions. Of these, 1004 are classified as pathogenic, while 691 are classified as benign. The MCC, F1, and AUC scores are similar between the holdout and test datasets (Fig. S6 and Table S5), indicating no overfitting of IndeLLM siamese, as both datasets

include indels that were not used at any point to train or re-adjust any of the original weights. The holdout dataset is included in our GitHub under the Analysis folder.

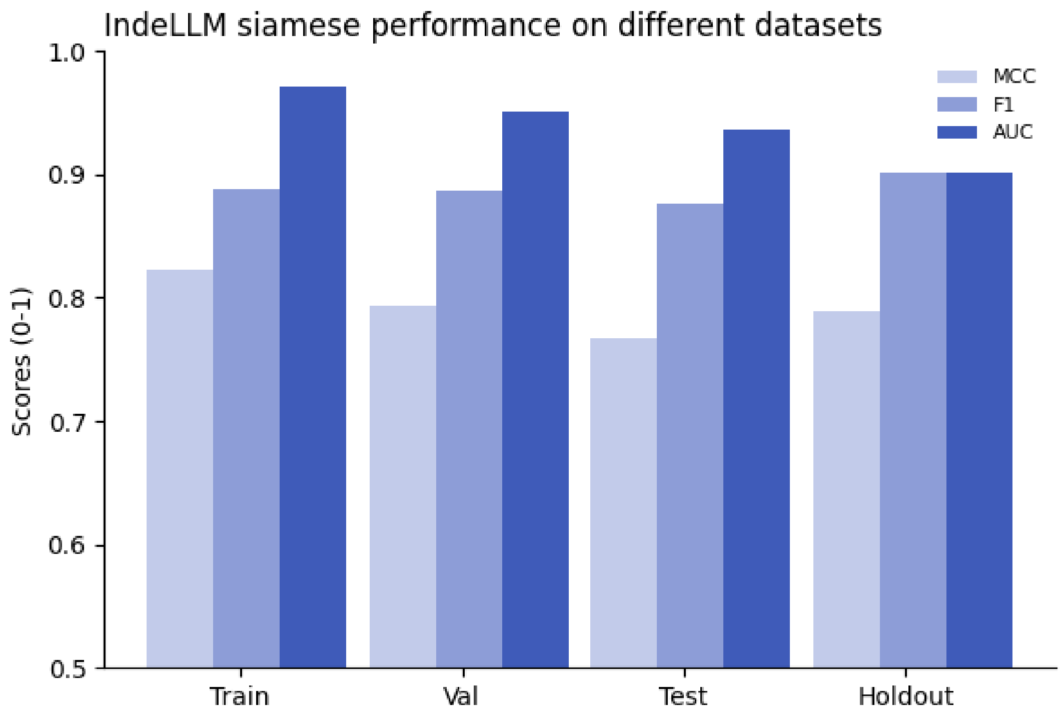

Figure S6 IndeLLM Siamese performance on different datasets: The MCC (light blue), F1 (blue) and AUC (dark blue) scores for the IndeLLM siamese using the training, validation, test and holdout dataset.

	**IndeLLM Siamese**		
**Datasets**	**F1**	**MCC**	**AUC**
Training	0.89	0.83	0.97
Validation	0.89	0.80	0.95
Test	0.88	0.77	0.94
Holdout	0.90	0.79	0.90

Table S5 IndeLLM Siamese performance on different datasets: The calculated MCC, F1 and AUC scores for IndeLLM siamese on the training, validation, test and holdout dataset.

S6. False negative insertions for IndeLLM and IndeLLM Siamese

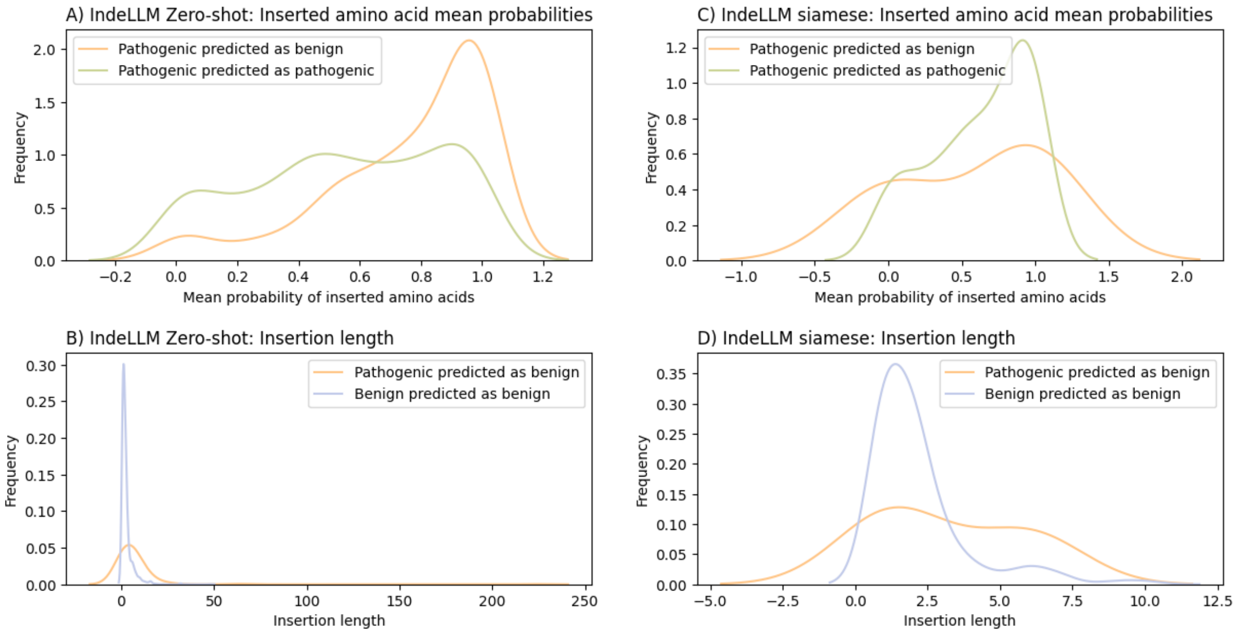

Figure S7 Length and probabilities distributions for the tested indels: Panels A and B show IndeLLM score calculations, while panels C and D are from IndeLLM Siamese. Panels A and C show the mean value of inserted amino acid, which was calculated for true positives (green) and false negatives (orange). In contrast, panels B and D show the length of inserted amino acids, which were calculated for false negatives (orange) and true positives (blue).

S7. AlphaFold prediction

To test our hypothesis that the 5 amino acid deletion in the Ig-like D1 domain of FGFR1 results in destabilisation of the domain, we ran the mutated sequence of the Ig-like domain (Uniprot ID: P11362 residue 37 to 125) on AlphaFold Collab [12]. All predictions from AlphaFold predict loss of a β-strand (Figure S7). The loss of this secondary structure element is most likely destabilising for the domain structure.

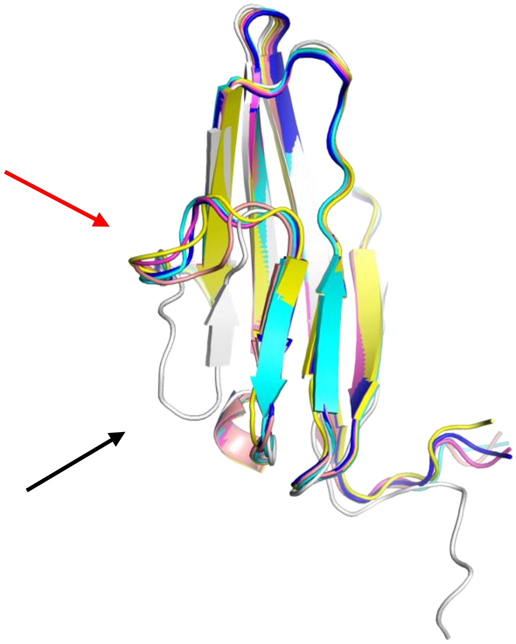

Figure S8 Visualization of Ig-like D1 domain of FGFR1 with different deletions: The wildtype Ig-like domain (PDB ID: 2CR3) superimposed on the five AlphaFold models (yellow, cyan, magenta, pink and blue). The black arrow highlights the lost β-strand in all predicted models (wildtype in white). The red arrow points to the truncated loop in the AlphaFold predictions.

S8. Computational efficiency

Provean is no longer supported (https://www.jcvi.org/research/provean), and the download is no longer compatible with today's systems. At the time of this analysis, the web server was accessible but allowed 100 indels runs per day, and processing of each indel takes 10-15 minutes if the sequences are not already downloaded in the server [13].

We downloaded the MutPred-Indel standalone to run the indels. We run all indels in batches of 100. The mean processing time per batch run was 4 hours and 35 minutes (2.75 minutes per job). However, some of this processing time is due to startup of the MATLAB dependency (http://mutpred2.mutdb.org)[14].

Non-multiple-sequence alignments (MSAs) Transformer-based approaches, as described in this work, are normally significantly faster than traditional methods that rely on multiple-sequence MSAs or data integration from multiple sources. One key advantage of transformers is their ability to process entire sequences in parallel using self-attention mechanisms rather than requiring iterative or sequential computations. This parallelisation allows transformer models to analyse protein sequences in batch, taking advantage of modern GPU and TPU architectures for high-throughput processing. A complete comparison of the performances of the two methods is out of the scope of this manuscript. However, using an RTX4000, IndeLLM was several orders of magnitude faster than the other methods that could be run locally.

References

1. Cannon, S., Williams, M., Gunning, A.C., and Wright, C.F. (2023). Evaluation of in silico pathogenicity prediction tools for the classification of small in-frame indels. BMC Med. Genomics *16*, 36. https://doi.org/10.1186/s12920-023-01454-6.

2. Fan, X., Pan, H., Tian, A., Chung, W.K., and Shen, Y. (2023). SHINE: protein language model-based pathogenicity prediction for short inframe insertion and deletion variants. Brief. Bioinform. *24*, bbac584. https://doi.org/10.1093/bib/bbac584.

3. Brandes, N., Goldman, G., Wang, C.H., Ye, C.J., and Ntranos, V. (2023). Genome-wide prediction of disease variant effects with a deep protein language model. Nat. Genet. *55*, 1512–1522. https://doi.org/10.1038/s41588-023-01465-0.

4. Meier, J., Rao, R., Verkuil, R., Liu, J., Sercu, T., and Rives, A. (2021). Language models enable zero-shot prediction of the effects of mutations on protein function. Preprint at bioRxiv, https://doi.org/10.1101/2021.07.09.450648.

5. Rives, A., Meier, J., Sercu, T., Goyal, S., Lin, Z., Liu, J., Guo, D., Ott, M., Zitnick, C.L., Ma, J., et al. (2021). Biological structure and function emerge from scaling unsupervised learning to 250 million protein sequences. Proc. Natl. Acad. Sci. *118*, e2016239118. https://doi.org/10.1073/pnas.2016239118.

6. Elnaggar, A., Heinzinger, M., Dallago, C., Rehawi, G., Wang, Y., Jones, L., Gibbs, T., Feher, T., Angerer, C., Steinegger, M., et al. (2022). ProtTrans: Toward Understanding the Language of Life Through Self-Supervised Learning. IEEE Trans. Pattern Anal. Mach. Intell. *44*, 7112–7127. https://doi.org/10.1109/TPAMI.2021.3095381.

7. Lin, Z., Akin, H., Rao, R., Hie, B., Zhu, Z., Lu, W., Smetanin, N., Verkuil, R., Kabeli, O., Shmueli, Y., et al. (2023). Evolutionary-scale prediction of atomic-level protein structure with a language model. Science *379*, 1123–1130. https://doi.org/10.1126/science.ade2574.

8. Hayes, T., Rao, R., Akin, H., Sofroniew, N.J., Oktay, D., Lin, Z., Verkuil, R., Tran, V.Q., Deaton, J., Wiggert, M., et al. (2024). Simulating 500 million years of evolution with a language model. Preprint at bioRxiv, https://doi.org/10.1101/2024.07.01.600583.

9. Hoffmann, J., Borgeaud, S., Mensch, A., Buchatskaya, E., Cai, T., Rutherford, E., Casas, D. de L., Hendricks, L.A., Welbl, J., Clark, A., et al. (2022). Training Compute-Optimal Large Language Models. Preprint at arXiv, https://doi.org/10.48550/arXiv.2203.15556.

10. Heinzinger, M., Weissenow, K., Sanchez, J.G., Henkel, A., Mirdita, M., Steinegger, M., and Rost, B. (2024). Bilingual language model for protein sequence and structure. NAR Genomics Bioinforma. *6*, lqae150. https://doi.org/10.1093/nargab/lqae150.

11. Notin, P., Kollasch, A., Ritter, D., van Niekerk, L., Paul, S., Spinner, H., Rollins, N., Shaw, A., Orenbuch, R., Weitzman, R., et al. (2023). ProteinGym: Large-Scale Benchmarks for Protein Fitness Prediction and Design. Adv. Neural Inf. Process. Syst. *36*, 64331–64379.

12. Mirdita, M., Schütze, K., Moriwaki, Y., Heo, L., Ovchinnikov, S., and Steinegger, M. (2022). ColabFold: making protein folding accessible to all. Nat. Methods *19*, 679–682. https://doi.org/10.1038/s41592-022-01488-1.

13. Choi, Y., and Chan, A.P. (2015). PROVEAN web server: a tool to predict the functional effect of amino acid substitutions and indels. Bioinformatics *31*, 2745–2747. https://doi.org/10.1093/bioinformatics/btv195.

14. Pejaver, V., Urresti, J., Lugo-Martinez, J., Pagel, K.A., Lin, G.N., Nam, H.-J., Mort, M., Cooper, D.N., Sebat, J., Iakoucheva, L.M., et al. (2020). Inferring the molecular and phenotypic impact of amino acid variants with MutPred2. Nat. Commun. *11*, 5918. https://doi.org/10.1038/s41467-020-19669-x.
